# AlphaFold2 models indicate that protein sequence determines both structure and dynamics

**DOI:** 10.1038/s41598-022-14382-9

**Published:** 2022-06-23

**Authors:** Hao-Bo Guo, Alexander Perminov, Selemon Bekele, Gary Kedziora, Sanaz Farajollahi, Vanessa Varaljay, Kevin Hinkle, Valeria Molinero, Konrad Meister, Chia Hung, Patrick Dennis, Nancy Kelley-Loughnane, Rajiv Berry

**Affiliations:** 1 Materials and Manufacturing Directorate, Air Force Research Laboratory, Wright-Patterson Air Force Base, 45433 OH USA; 2 UES Inc., Dayton, OH USA; 3 Computer Science Department, Miami University, Oxford, OH USA; 4 General Dynamics Information Technology, Inc., Wright-Patterson Air Force Base, 45433 OH USA; 5 Department of Chemical and Materials Engineering, Dayton University, Dayton, OH USA; 6 Department of Chemistry, The University of Utah, Salt Lake City, UT USA; 7 Department of Natural Sciences, University of Alaska Southeast, Juneau, AK USA; 8 Max Planck Institute for Polymer Research, Mainz, Germany

**Keywords:** Protein folding, Protein function predictions, Protein structure predictions

## Abstract

AlphaFold 2 (AF2) has placed Molecular Biology in a new era where we can visualize, analyze and interpret the structures and functions of all proteins solely from their primary sequences. We performed AF2 structure predictions for various protein systems, including globular proteins, a multi-domain protein, an intrinsically disordered protein (IDP), a randomized protein, two larger proteins (> 1000 AA), a heterodimer and a homodimer protein complex. Our results show that along with the three dimensional (3D) structures, AF2 also decodes protein sequences into residue flexibilities via both the predicted local distance difference test (pLDDT) scores of the models, and the predicted aligned error (PAE) maps. We show that PAE maps from AF2 are correlated with the distance variation (DV) matrices from molecular dynamics (MD) simulations, which reveals that the PAE maps can predict the dynamical nature of protein residues. Here, we introduce the AF2-scores, which are simply derived from pLDDT scores and are in the range of [0, 1]. We found that for most protein models, including large proteins and protein complexes, the AF2-scores are highly correlated with the root mean square fluctuations (RMSF) calculated from MD simulations. However, for an IDP and a randomized protein, the AF2-scores do not correlate with the RMSF from MD, especially for the IDP. Our results indicate that the protein structures predicted by AF2 also convey information of the residue flexibility, i.e., protein dynamics.

## Introduction

The protein sequence-structure gap had been observed for decades^1,2^. The success of AlphaFold2 (AF2) promises to fill this gap by predicting protein structures with experimental accuracy based solely on their primary amino acid sequences^3,4^. In a recent special issue published in the Journal of Molecular Biology, AF2 was highlighted as “a once-in-a-lifetime breakthrough in science”^5^, because AF2 successfully demonstrated that the structure of a protein can be determined based on its sequence using modeling. We are now in a position to demonstrate Anfinsen’s^6^ proposal: a protein’s sequence determines its structure, which in turn determines its function. Inspired by AF2, other tools have been announced, including RoseTTAFold^7^ and AlphaFold-Multimer (a recent update of AF2)^8^ which are sufficient to describe three-dimensional (3D) protein–protein interactions (PPIs) in the cells^9^. These tools have opened new avenues in biology and medicine^10,11^ and have shown promise in protein engineering and biodesign^12–15^.

Despite the exciting progress, challenges persist, demanding further development of AF2 and other algorithms, to model membrane proteins^16^, intrinsically disordered proteins (IDPs)^17–19^, misfolded and aggregated proteins^20–22^, glycoproteins^23^, large-size proteins^4^ and protein complexes^24^, as well as other proteins belonging to the dark proteome^25,26^. Nevertheless, compared to homology modeling methods^27,28^, the deep learning-based AF2 method has established the potential of constructing protein structures of whole organismal proteomes^4^, i.e., the *structurome*, with near-experimental accuracies. To better utilize the AF2 models, it is of considerable importance to understand how these models have been built and evaluated by AF2, i.e., what are the AF2 model construction procedures and its evaluation metrics? In this report we provide insight into the evaluation metrics, connecting them to protein structural dynamics.

Starting from a multisequence alignment (MSA), AF2 uses the Evoformer network that integrates both biological and physical information contained in its databases^3^. The structural module of AF2 also creates the model confidence predictions, reported as the predicted local distance difference test (pLDDT) scores^3,29,30^. The pLDDT scores are in the range of [0, 100]. High pLDDT scores (e.g., > 80) indicate high confidence of the residue structure, and low pLDDT scores (e.g., < 50) may indicate that the residues are in intrinsically disordered protein regions (IDPRs)^19,31,32^. Another useful metric to qualify/quantify the structure prediction is the global superposition TM-score^33^. AF2 calculates the predicted template modeling, or TM-scores (pTM) based on a pairwise error prediction, and is used to calculate the predicted aligned error (PAE) that estimates the error of the position of each amino acid^3^. It was shown that the MSA depth from the protein sequence strongly affects prediction accuracy^3^. It was also established that using MSA alone is sufficient for fast and effective protein structure predictions^34^.

Proteins are not static, their configurations constantly change and they frequently make interactions with other molecules in the cell. The models that AF2 generates only represent single snapshots of the proteins, and AF2 developers suggest multiple runs to properly sample model configurations to represent biological diversity^3^. In reality, the users may only consider the best-ranked model (i.e., the model with the highest overall pLDDT scores) for further analysis. Both pLDDT scores and PAE matrices are estimated from a comparison of the predicted structure and the “real” (ground truth) structure in AF2 reports. However, the original AF2 manuscript recognized that there are actually no “real” structures^3^. The proteins perform their functions through interactions and movements, especially in the crowded cell environment. The diversities in structure models or protein movements are related to protein dynamics. Therefore, it is important to ask if AF2 can also predict the dynamic characters, or dynamics personalities, of proteins^35^, which adds the fourth dimension of time of all atoms in a protein.

In the present work, we suggest an answer to this question. By comparing molecular dynamics (MD) simulations with the AF2 predictions, we show that AF2 not only predicts the protein 3D structure, but also gives clues about the protein dynamics, via both the pLDDT scores and PAE matrices. We compared the flexibility scores from MD (root-mean-square fluctuation, or RMSF) with the pLDDT scores reported by AF2, as well as the intrinsic disorder contents predicted by IUPRED2^36^. We found that for proteins with high MSA depth the pLDDT scores highly correlate with the RMSF scores. We also calculated the one-dimensional (1D) distance variations (DV) between the Cɑ carbons, and found that the DV matrix from MD is highly consistent with the PAE matrix from AF2, indicating that the PAE matrix originates from protein dynamics. We further tested an intrinsically disordered protein and a randomly constructed protein, whose sequences were found to have no MSA hits. In these cases, the pLDDT scores from AF2 poorly predict residue flexibilities. Therefore, in AF2 modeling, biological information through multisequence alignment, may not only be translated to structural information, but also contains other biophysical information, including information about which residues are mobile. Such information provides valuable insights into protein dynamics and clues about how they function.

## Methods

### Protein structure models

AF2 (V2.0.1) is used for structure predictions with the required databases downloaded from the AF2 GitHub repository^3^. Table 1 summarizes the protein models used in the present work. The AF2 structure models of these proteins are shown in Fig. S1 of the Supplementary Information (SI). All protein sequences can be found in the Appendix of the SI.

**Table 1:** Proteins models used in the present work.

Protein	AA^1^	MSA^2^	pLDDT^3^	IUPRED2^3^	RMSF (Å)^3^	PCC^6^	Slope^6^	Int.^6^
a. LanM	133	1832	83.9 ± 19.1	0.39 ± 0.16	5.8 ± 3.3	− 0.84	− 4.9	113
b. DeHa4	300	1890	96.3 ± 6.9	0.25 ± 0.14	0.9 ± 0.9	− 0.94	− 7.2	103
c. PAS-A Domain	108	1138	81.4 ± 16.3	0.20 ± 0.09	1.0 ± 0.7	− 0.65	− 15.5	97
d. AFP Type III	66	1080	96.4 ± 5.7	0.20 ± 0.07	0.7 ± 0.7	− 0.97	− 8.4	103
e. GNE	722	5273	93.2 ± 11.4	0.21 ± 0.12	3.0 ± 1.1	− 0.75	− 9.6	105
f. PAS-Kinase	1323	8644	52.9 ± 27.5	0.43 ± 0.25	5.0 ± 3.9	− 0.63	− 4.0	77
g. inaZ	1200	2050	88.6 ± 16.5	0.41 ± 0.07	3.8 ± 3.2	− 0.65	− 3.3	101
h. Heterodimer^4^: PAS-A, kinase	108 287	1138 1908	89.5 ± 13.0	0.14 ± 0.10	1.3 ± 0.7	− 0.65	− 11.7	110
i. Homodimer^5^: MtMerR	146 146	1825 1825	89.3 ± 13.9	0.36 ± 0.13	3.8 ± 2.5	− 0.66	− 3.7	103
j. NVJP-1	388	0	43.2 ± 5.3	0.84 ± 0.13	10.2 ± 2.4	− 0.03	− 0.1	44
k. Randomized	237	0	32.4 ± 6.2	0.28 ± 0.19	2.1 ± 1.1	− 0.12	− 0.7	34

^1^Number of amino acid residues.

^2^The MSA hits from the BFD^3^ (Big Fantastic Database). The MSA hits include those that match the protein partial segments.

^3^Mean ± SD for per-residue pLDDT, IUPRED2 and RMSF values.

^4^Two chains of the heterodimer are PAS-A (108 AA) and kinase (287 AA) domain sequences, respectively.

^5^Both chains of the homodimer have the same sequence of 146 AA.

^6^The Pearson’s correlation coefficient (PCC) between pLDDT and RMSF scores, the slope and intercepts of the linear fitting between them are also listed; note that as pLDDT and the AF2 scores in this work are anticorrelated, and the PCC values are the negative of those shown in the Figures.

### MD simulation

Molecular dynamics (MD) simulations were performed using the NAMD program^37^. The protein atoms were typed according to the CHARMM force field^38,39^ (c36m) and a modified TIP3P model^40^ was used for the solvent water molecules. All hydrogen atoms were added using the HBuild function of CHARMM^40,41^. The proteins were placed in unit cells with at least 12 Å of solvent water molecules added to each edge. The solvation and neutralization (using Na^+^ and/or Cl^−^) were carried out by the Solvate and Autoionization packages of VMD^42^. After solvation and neutralization, the whole system was optimized by 50,000 steps. Next, the temperature of the system was increased to 300 K with a rate of 0.001 K/timestep, followed by constant-pressure, constant-temperature (NPT) equilibration at a pressure of 1 atm and temperature of 300 K maintained by Langevin piston controls.The SHAKE algorithm was applied to fix the bond lengths involving hydrogen atoms. The simulations were conducted using a timestep of 2 fs and a non-bonded interaction cutoff switching of 9 to 11 Å. The protonation states of all titratable residues of the protein were determined by ProPka^43^ at neutral pH of 7. For each protein, the system was equilibrated for 10 ns, followed by a 100 ns production run with the trajectory saved every 10 ps. The analyses were based on the production runs (10 k frames each).

### Analysis of the MD trajectories

We used the R package bio3d^44,45^ to analyze the 100 ns production run MD trajectories: the root mean square deviation (RMSD), root mean square fluctuation (RMSF) calculations and the mass-weighted principal component analysis (PCA) for the movement of the protein backbone atoms.

**Figure 1:**
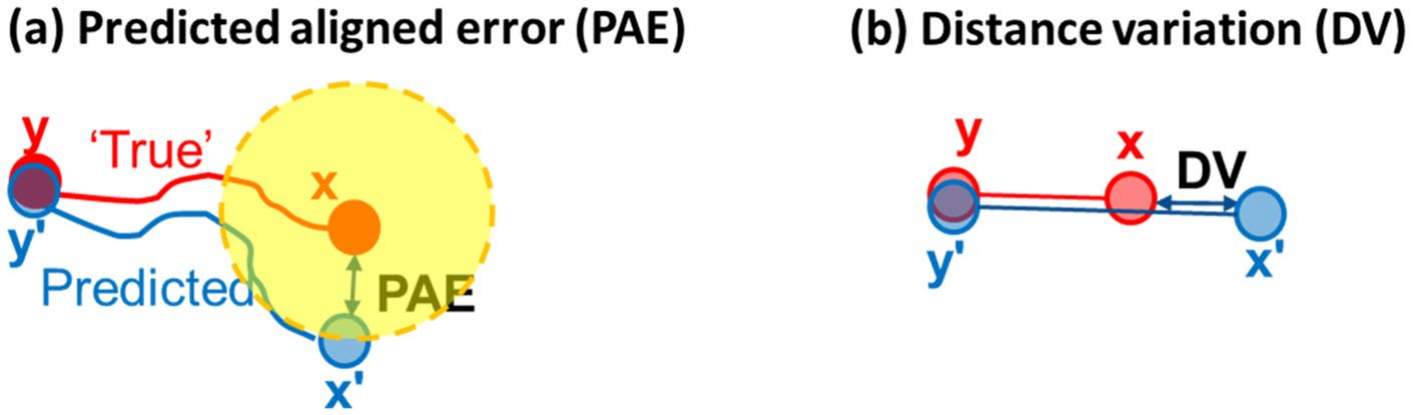
PAE vs DV. (**a**) PAE(x,y) is the error of residue x between the predicted (blue) and the “true” (red) models when residue y is aligned (x’ could be observed anywhere in the yellow sphere). (**b**) DV(x,y) is the distance variation between residues x and y monitored from MD, and the DV can be regarded as the (1D) variations in the movements of residue x with the position of residue y fixed.

Distance variations (DV) were calculated from the MD simulations in order to gain insight into the predicted aligned errors (PAE) provided by AF2.. With the assumption that the PAE map contains the dynamics information of the protein, the DV can be regarded as a 1D simplification of the PAE (see below). In the definition and estimation of the PAE, AF2 performs “alignments” between the predicted structure and the “true” (or “real”) structure. The PAE between the residues x and y, i.e., the (x, y) element of the PAE matrix is estimated as the error of the x residue if the y residue is aligned (Fig. 1)^3,29^. Here, for two residues x and y, we define the distance deviation (DV) as:

1 $$ DV = IQR\left( {r_{xy} } \right), $$

where *r*_*xy*_ is the distance between the Cɑ atoms of residues x and y monitored through the MD trajectory. IQR is the interquartile range. The DV also has a unit of Å. If we assume the residue y is fixed, then the calculated IQR could be regarded as the (1D) variation of residue x. Use of IQR in Eq. (1) can avoid the biases from outliers (extreme long or short *r*_*xy*_). Note that the PAE matrix is asymmetric^3,29,46^ as for any (x, y) pair, the uncertainty assigned to x may be different than that to y. However, the DV matrix is symmetric because the distance variations neglected the residue compositions. We consider the dynamic assumption valid if the DV map matches the PAE map—that is, the PAE map from AF2 originates from the protein dynamics.

### Other tools and data availability

Besides AF2 structure prediction and the MD simulations, IUPRED2^36^ was used for prediction of the intrinsic disorder content based on the protein sequence. VMD^42^ was used to plot structural figures and generate animations of the principal movement (PC1) of the proteins during the MD simulations. PyMol (2.4.1) was used to plot the residue dynamic cross correlation matrix (DCCM) analyzed by bio3d^44^. All figures were prepared using R. The R codes used for the principal component analysis, and the DV calculations (together with the heatmap plot) are available from the GitHub repository: https://github.com/haoboguo/AF2.Res.Flex. This repository also contains the coordinates (PDB format) of the AF2 structures used in this work, and the animations of the primary movements (PC1) of the two domain protein GNE (vibration between the two domains) and the homodimeric MerR-family protein (opening-and-closing dynamics) from *Mycobacterium tuberculosis*, calculated by PCA. All heatmaps were plotted using the heatmap.2 function from the gplots package of R.

The B-factors from X-ray crystallography were used when available. Because the B-factor can be compared with RMSF via,

2 $$ B = \left( {8\pi^{2} /3} \right)RMSF^{2} , $$

where RMSF is derived from MD: a higher *B* or *RMSF* value corresponds to higher flexibility of a residue. Similarly, the B-factors could be inferred from an ensemble of configurations detected by NMR^47^. The square root of B-factors was used in the comparisons with RMSF (see in “Results and Discussion”).

**Figure 2:**
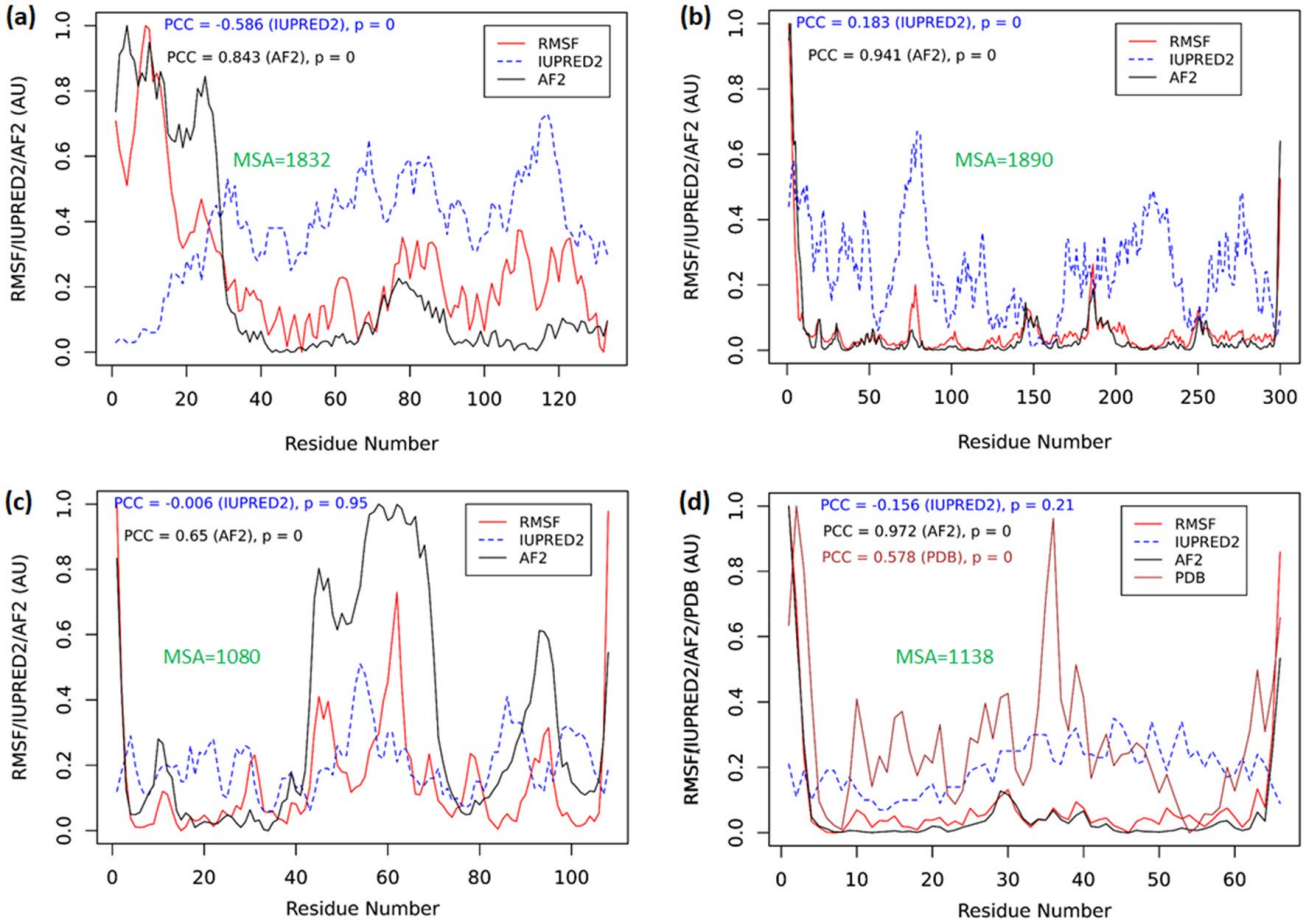
The AF2 scores predict the residue flexibility. The RMSF (red), AF2 (black), IUPRED2 (blue dashed) scores, and the B-factor from X-ray crystallography (brown, PDB score) in (**d**) are plotted. Arbitrary units (AU) are used as the RMSF, AF2 and PDB scores are all normalized and unitless. (**a**) Lanmodulin (LanM); (**b**) Dehalogenase; (**c**) PAS-A domain protein (**d**) Antifreeze protein with known X-ray crystal structure (PDB ID: 1HG7).

The pLDDT scores are listed in the B-factor column of the AF2 protein models in the AlphaFold database^4,46^. However, we found the pLDDT scores from the AF2 protein models (Fig. 2) usually show an anti-correlation with the RMSF values calculated from MD. Furthermore, the pLDDT scores exhibit an opposite trend to what the B-factors or RMSF indicate. Here, for consistency, we define a new parameter, the AF2-score, as the normalized reversed pLDDT scores, i.e.,

3 $$ AF2 - score = \left( {pLDDT_{max} - pLDDT} \right)/\left( {pLDDT_{max} - pLDDT_{min} } \right). $$

The calculated AF2-scores are highly correlated with the RMSF (see Results and Discussion section), indicating the models generated by AF2 also contain information about the residue flexibility. For PAE and DV heatmaps, the color gradually changes from the highest (white) to lowest (dark green) in 256 bins, only exception is for the DV heatmap of PAS-Kinase, the color bar is manually adjusted (Fig. 4 caption) to highlight the similarity of both matrices.

## Results and discussion

### AF2-scores represent residue flexibility

AF2 provides the per-residue pLDDT (predicted local distance difference test) scores for each residue of the final model. This score is in the range of [0,100] and represents the confidence of the predicted structure compared to the “true” (ground truth) structure. We used the AF2-scores, as a reversed normalization of the pLDDT scores (see Methods section), and found that the AF2-scores are highly correlated with the residue flexibility. In reality, even the “true” X-ray crystallographic structure represents an ensemble of protein configurations embedded in the crystal lattices. The flexibilities of the atoms are usually recorded as the temperature factors (or B-factors) in the PDB files. From MD simulations, the residue flexibility can be calculated as the root-mean-square fluctuation (RMSF, see “Methods”).

Figure 2 compares various measures related to residue flexibility for the AF2 models built from the sequences of four proteins. The first protein (Fig. 2a, 133 AA) is the apo-form of the full length lanmodulin (LanM) protein. LanM is an interesting protein that could shed light on rare earth element sequestration^48–50^. The LanM protein solved by NMR binds to three yttrium ions and has its N-terminus (residues M1 to A22) cleaved^51^ (PDB ID 6MI5). For the apo LanM, MD simulation shows high-flexibility of the N-terminal tail, consistent with the AF2-scores. The RMSF of all residues is highly correlated with the AF2-scores (Pearson’s correlation coefficient, or PCC = 0.843, *P* = 0). However, the disorder prediction by IUPRED2 interprets that the N-terminus is in a well-ordered state (the median disorder content of 0.07), contradicting both the AF2 score and the RMSF calculated from MD.

The second protein studied (300 AA) is a dehalogenase recently sequenced from the bacterium *Delftia acidovorans strain D4B*^52^. Because *D. acidovorans* had been cultured in presence of perfluorooctanoic acid (PFOA)^52^, this dehalogenase might be involved in defluorination of PFOA (or other fluorinated compounds). For this model, the RMSF from MD correlates well with the AF2-score for the dehalogenase (PCC = 0.941, *P* = 0), as shown in Fig. 2b. Moderate correlation is observed between RMSF and the IUPRED2 scores (PCC = 0.183, *P* = 0).

The third protein is part of the human PAS-A-domain containing serine/threonine kinase (1323 AA, Q96RG2 in the AF2 database). Here the PAS-A domain sequence (M130-R237, M130 is mutated from the original P130) is used to build the PAS-A domain model. The PAS-A domain is speculated to regulate the kinase activity by sensing environmental stimuli. In general, PAS domains are found in all three domains of life and have a well-conserved tertiary structure, albeit with diverse sequences^53^. It is shown in Fig. 2c that the high flexibility of the central region of the PAS-A domain (residues 45 to 70) revealed by the RMSF profile is also reproduced by the high AF2-scores (low pLDDT scores). However, the IUPRED2 score does not correlate with the RMSF for this protein.

The fourth protein is an antifreeze protein (AFP, Fig. 2d, 66 AA), which also has a high-resolution X-ray crystallographic structure (PDB entry 1HG7^54^, resolution 1.15 Å), such that the experimental B-factors are available for comparisons. In this example, the AF2 score has a near-perfect correlation with the RMSF (PCC = 0.972, *P* = 0). However, the IUPRED score does not show positive correlation with the RMSF. The crystal lattice effect in the X-ray structure may lead to rigid body motions which affect the B-factor profile (square-root used, see Eq. 2), but it also shows good correlation with the RMSF (PCC = 0.578, *P* = 0).

The data for all four models in Fig. 2 indicate that the AF2 scores can be used to predict the residue flexibilities, as measured by the RMSF from MD simulations. However, for these proteins, the disorder predictors (such as IUPRED2) for these proteins do not seem to predict residue flexibility. It had been shown that combining both the flexibility (B-factor) and the disorder contents the protein sequences can be classified into four different categories: low-B-factor ordered, high-B-factor ordered, short-disordered and long-disordered regions^55^. This also explains why the IUPRED2 scores and the RMSF values do not correlate well. The above results indicate that in addition to solving 3D structures from amino acid sequences, AF2 accurately predicts residue flexibilities from the pLDDT scores (or AF2-scores). It should be pointed out that all protein sequences in Fig. 2 have relatively large MSA depths (> 1000)一here, the MSA depth is defined as the number of aligned or partially aligned sequences from the BFD^3^ (see Table 1 for the MSA depth of all models used in the present work).

### PAE from AF2 is associated with protein dynamics

The predicted aligned errors (PAE) derived from the predicted template modeling (pTM) scores clearly show that the residues within the same domain exhibit lower PAEs than the inter-domain residues. The AF2 model that serves as the tutorial for the PAE is the human GNE protein, a two-domain, bifunctional enzyme playing a key role in sialic acid biosynthesis^56^. Using this model structure, an MD simulation was performed and the distance variations (DVs) among the Cɑ atoms of residues were computed to compare with the PAE map.

**Figure 3:**
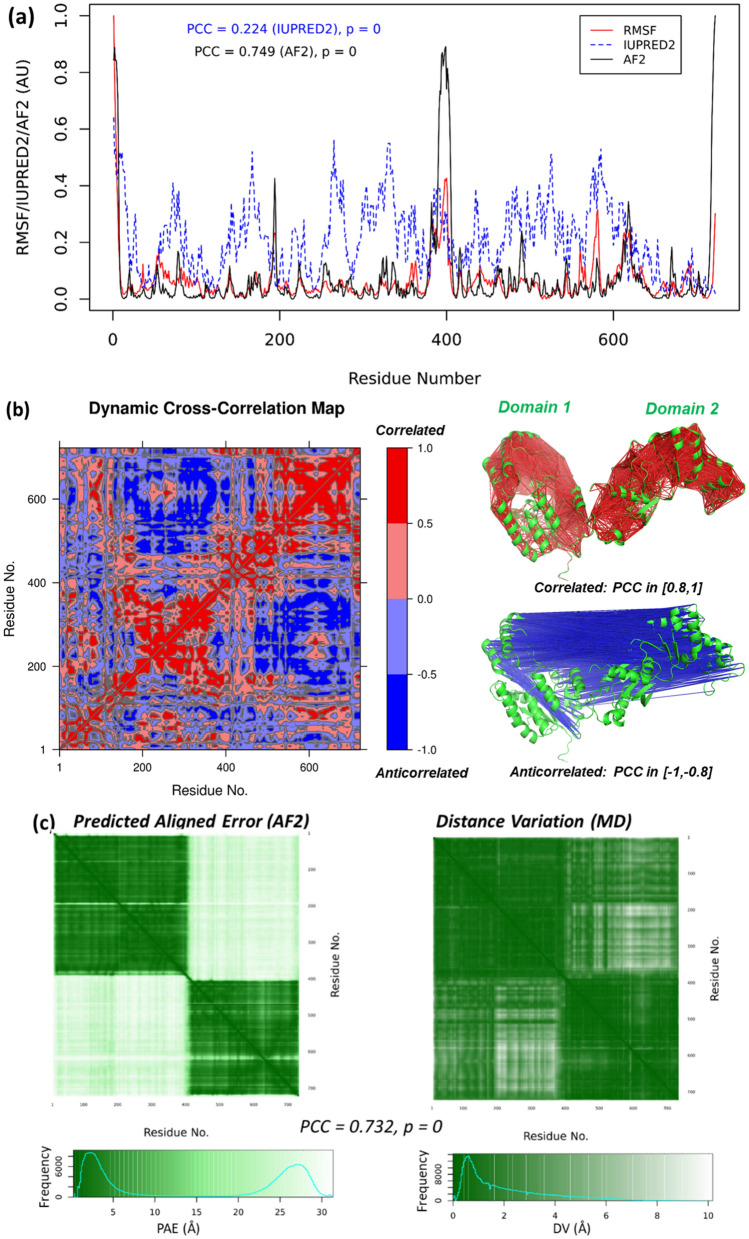
The dynamic nature of the predicted aligned error from AF2 and the dynamics of a two-domain protein GNE (AF2 entry: Q9Y223). (**a**) The AF2 scores correlate well with the RMSF values calculated with a domain-specific approach (see in main text); the IUPRED2 scores also show a correlation to the RMSD. An arbitrary unit (AU) is applied as both AF2 and RMSF are normalized. (**b**) Residue cross correlations calculated from the MD trajectory show that residues within the same domain tend to have correlated movements (i.e., moving toward the same directions) whereas residues from different domains tend to have anticorrelated movements (or moving toward opposite directions). This is also reflected in the principal movement, which is the vibration between the two domains (Supplementary movie). (**c**) The predicted assigned error (PAE) map is calculated via AlphaFold2-pTM and it can also be found in the AF2 PAE tutorial (the coloring scheme is slightly different) (https://alphafold.ebi.ac.uk/entry/Q9Y223); and the distant variation (DV) map calculated from a 100-ns MD trajectory. Both heatmaps indicate that the residues within the same domain have a relatively small PAE (left) or DV (right), whereas the PAE/DV for residues from different domains are relatively large. The color histograms of the PAE or DV values are also plotted in the color bars: Both PAE and DV histogram have a peak at short distances, but in PAE histogram there is an additional peak at long distance (ca. 27 Å), indicating AF2 yields larger interdomain errors than the MD simulation.

For multi-domain systems, all-atom structural superposition based on the minimization of the overall RMSD^57^ may be inappropriate. For these systems, a principal component analysis can be used to examine the relationship between the domains^58^. The AF2 profile of the two domain GNE protein (Fig. 3a) shows that the linker (residue 381 to 401) between the two domains has high AF2-scores, together with both the C- and N-termini, indicating high flexibility of these regions. Instead of all-atom structural superposition, we used a domain-specific approach: first, only the residues of domain 1 (1 to 380) were superimposed and the RMSF values for domain 1 were acquired based on this superposition; then only the residues of domain 2 (402 to 722) are superimposed and the RMSF values for domain 2 calculated. However, the RMSF values of the linker region (residues 381 to 401) were averaged from both superpositions. This domain-specific superposition approach yielded RMSF of the whole protein highly consistent with the AF2-scores (Fig. 3a). This analysis is in line with our hypothesis that AF2 correctly predicts the residue flexibility via the pLDDT or AF2-scores (Fig. 2). We also calculated the RMSF values using an all-atom superposition approach as those for the one-domain proteins (Fig. 2), however, this approach cannot correctly address the flexibility, especially that of the linker between the domains (Fig. S2 in the SI). The RMSF profile of the LanM protein shown in Fig. 2a is obtained from an all-atom superposition. Similar to the domain-specific approach used for GNE, because LanM has a long disordered N-terminus (residues 1–22), if we average the RMSFs from superposition with or without the N-terminus (residues 23 to 133), the AF2-scores have a better correlation to the RMSF plot, as shown in Fig. S3 in the SI.

MD simulation studies often use the RMSD (root mean square deviation) profile with respect to the first frame of the trajectory, to determine whether the system has equilibrated. However, in the two-domain system, we observed that the principal movement (PC1 from PCA) corresponds to the anti-correlated movement of the two domains (Fig. 3b and Supplementary movie). With the large amplitude interdomain movement, the RMSD profile of this protein does not converge to a plateau. This has been observed previously for the MerR homodimer system regarding opening-and-closing dynamics^59^. We also monitored the interdomain center-of-mass (COM) distances from the MD trajectory, observing large amplitude fluctuations during the 100 ns MD with the interdomain COM varying from 47.5 to 60 Å (Fig. S4 in the SI). Interestingly, the RMSD profiles monitored using the two extreme interdomain COM configurations as the reference states exhibited mirror symmetry owing to the anticorrelation of the RMSD profiles. This hidden symmetry from MD simulations of vibrating systems had also been observed in both MerR and CurR homodimer proteins^59^.

We calculated the distance variations (DV) among the Cɑ atoms of all residues. The DV map and PAE map from AF2 (Fig. 3c) show highly consistent patterns: the distance variations (or predicted errors) of residues within the same domain are relatively small; whereas the variations/errors of residues from different domains are relatively large. For this two domain protein, the maximal PAE reported from AF2 is 31.8 Å, and the maximal calculated DV is 18.0 Å. This may be due to that the DV is estimated as the IQR, i.e., the Cɑ-Cɑ distance at the 75% quantile subtract that at the 25% quantile, which may be roughly half of difference between maximal and minimal Cɑ-Cɑ distance. Also the DV calculation is a 1D simplification of the real PAE (Fig. 1), which may also give errors to the estimation. However, the consistent trends between PAE and DV maps (PCC = 0.732, *P* = 0, Fig. 3c) indicate that PAE originates from protein dynamics, and that the Evoformer neural network of AF2 decodes this dynamics information (encoded in the protein sequences) through multisequence alignment.

The PAE and DV heatmaps for the models in Fig. 2 (see Fig. S5) show statistically significant correlations similar to Fig. 3c. These results substantiate the usefulness of the PAE’s predicted by AF2 for capturing protein dynamics.

### Large proteins

It remains a challenge for AF2 to model extremely large proteins, such as the human *Titin* protein (34,350 AAs) which include a long IDPR of over 2100 AAs^60^. The AlphaFold database of the human *structurome* does contain 3D models for smaller fragmental (1400 AA) of the *Titin*^4^. Other proteins containing residues as large as 2,180 AA’s (with no structural homologues) have also been reported with the TM-score of 0.96^3^. Here, we have also modeled two large proteins (> 1000 amino acids), including one with considerable disordered regions.

**Figure 4:**
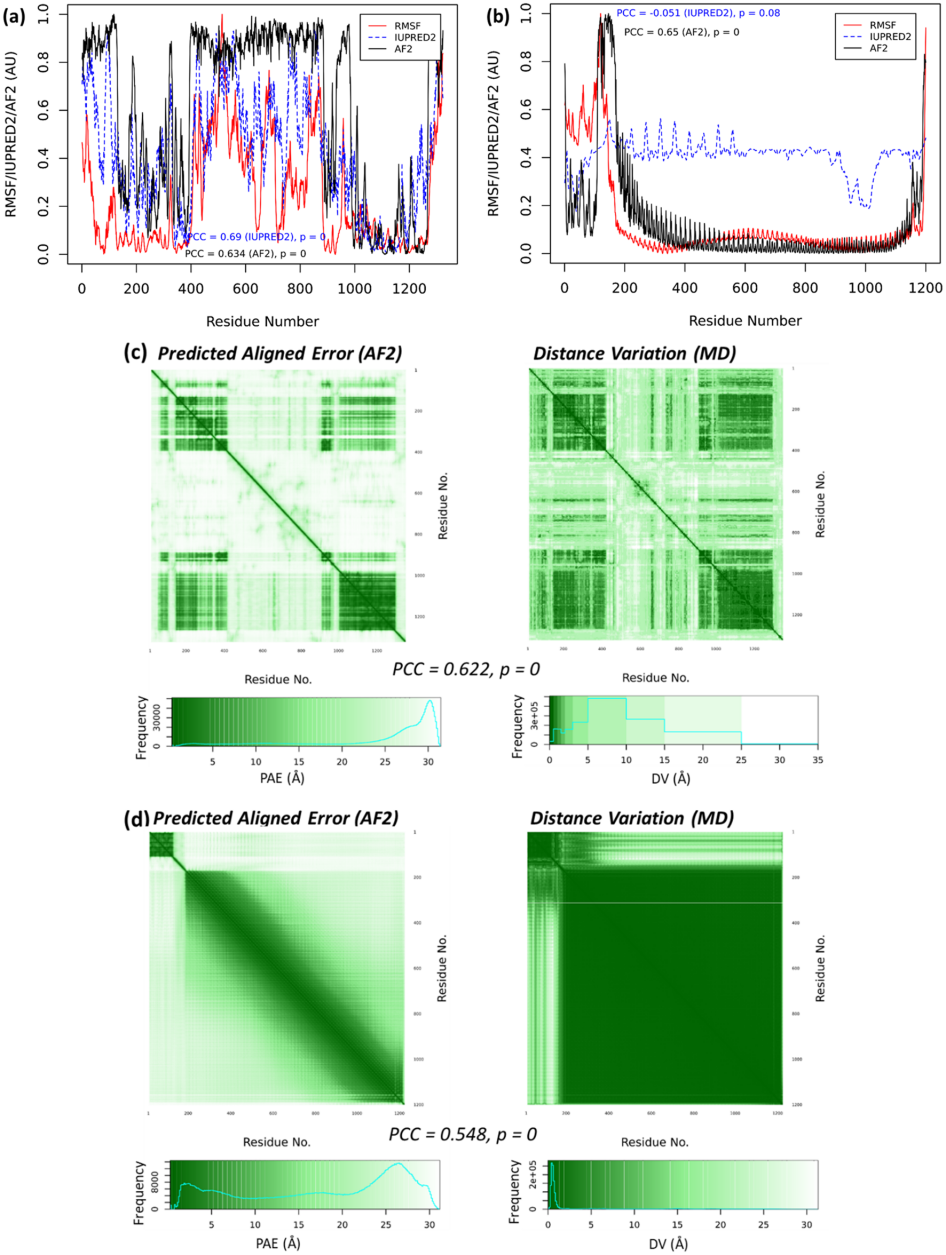
Large proteins. The RMSF, the AF2 scores, and IUPRED2 scores of (**a**) PAS-kinase and (**b**) ice nucleation protein inaZ. An arbitrary unit (AU) is used as both AF2 scores and RMSF are normalized. The PAE and DV maps of (**c**) PAS-kinase and (**d**) inaZ. In the DV heatmap of PAS-kinase (**c**, right), the color bar breaks (unit in Å) are set at (0, 0.5, 1, 1.5, 2, 3, 5, 10, 15, 25 35) to capture the similarity of both PAE and DV matrices (PCC = 0.622, *P* = 0).

Figure 2c represents the result for the PAS-A domain protein, which is only the regulatory part of the PAS-A domain containing kinase (PAS-kinase) from *H. sapiens*^53^. We modeled the structure of the full length PAS-kinase, which contains 1,323 AAs. This structural model has been also modeled by the AF2 team^4^ (AF2 entry: Q96RG2) and can be obtained from the AF2 database at (https://www.alphafold.ebi.ac.uk/entry/Q96RG2). In the present work, both the AF2-scores and the PAE map (Fig. 4a) indicate the PAS-kinase model indicates two structural domains: the PAS-domain that comprises both the PAS-A domain (residue 130 to 237, Fig. 2c) and PAS-B domain (residue 238 to 401) as well as the kinase-domain (residues 892 to 1269). However, the other regions of the full PAS-kinase are mostly disordered (see the PAS-kinase structure in Fig. S1). Both the AF2 and IUPRED2 scores correlate well with the RMSF calculated from the MD simulation (Fig. 4a). The PAE map also indicates the existence of two structure regions (or domains): the PAS-domain and the kinase domain, which is also supported by the DV map (Fig. 4c). Moreover, the interdomain regions in the DV map have relatively small distance variations, which is consistent with the PAE map and reflects the interactions between the two domains.

We also analyzed the ice nucleation protein *inaZ* from *Pseudomonas syringae* (UniProt code P06620). This 1,200 amino acid protein enables the microbial organism to facilitate the crystallization of supercooled water^61^. The ice nucleating properties of *P. syringae* are key for their biological function^62,63^, and confer them a role in cloud glaciation and precipitation^64,65^, as well as in artificial snow making^66^. Fragments of the inaZ protein had been modeled^67^ but the structure of the full-length protein has never been predicted. The AF2 structure (Fig. S1 in the SI) indicates that the inaZ has a N-terminal domain in ɑ/β fold (residues M1 to A110) and a ca. 30 nm-long domain constituted by antiparallel β-strands (residues Q171 to K1189), followed by C-terminal tail in coil state (residues P1180 to K1200).

For the inaZ protein, the AF2-scores are also strongly correlated with the RMSF from the MD simulation (Fig. 4b). The PAE map from AF2 and DV map from MD (Fig. 4d) both indicate the existence of two separated segments in the inaZ protein. The AF2 profile (Fig. 4c) shows repeating peaks from the β-strands, which is also reflected in the RMSF profile. Although the magnitudes of these peaks are different, the peak positions are precisely consistent. This consistency is also shown in other systems (Figs. 2 and 3). For the other large protein PAS-kinase (Fig. 4a), overall correlation between AF2 and RMSF profiles is observed, but not at the finger-print-like accuracy of inaZ, which may be owing to the IDPRs in the PAS-kinase (Fig. S1 in the SI). Similarly, the PAE and DV maps are also consistent, but the PAE from AF2 modeling generally propose larger error ranges than the DV from MD. Not only because DV can be regarded as a 1D simplification as PAE (see Methods), this may also be derived from the PAE evaluation, which is based on the calculation of the predicted template modeling scores of the predicted residues compared to the imaginary “true” models^3^.

### Multimers

**Figure 5:**
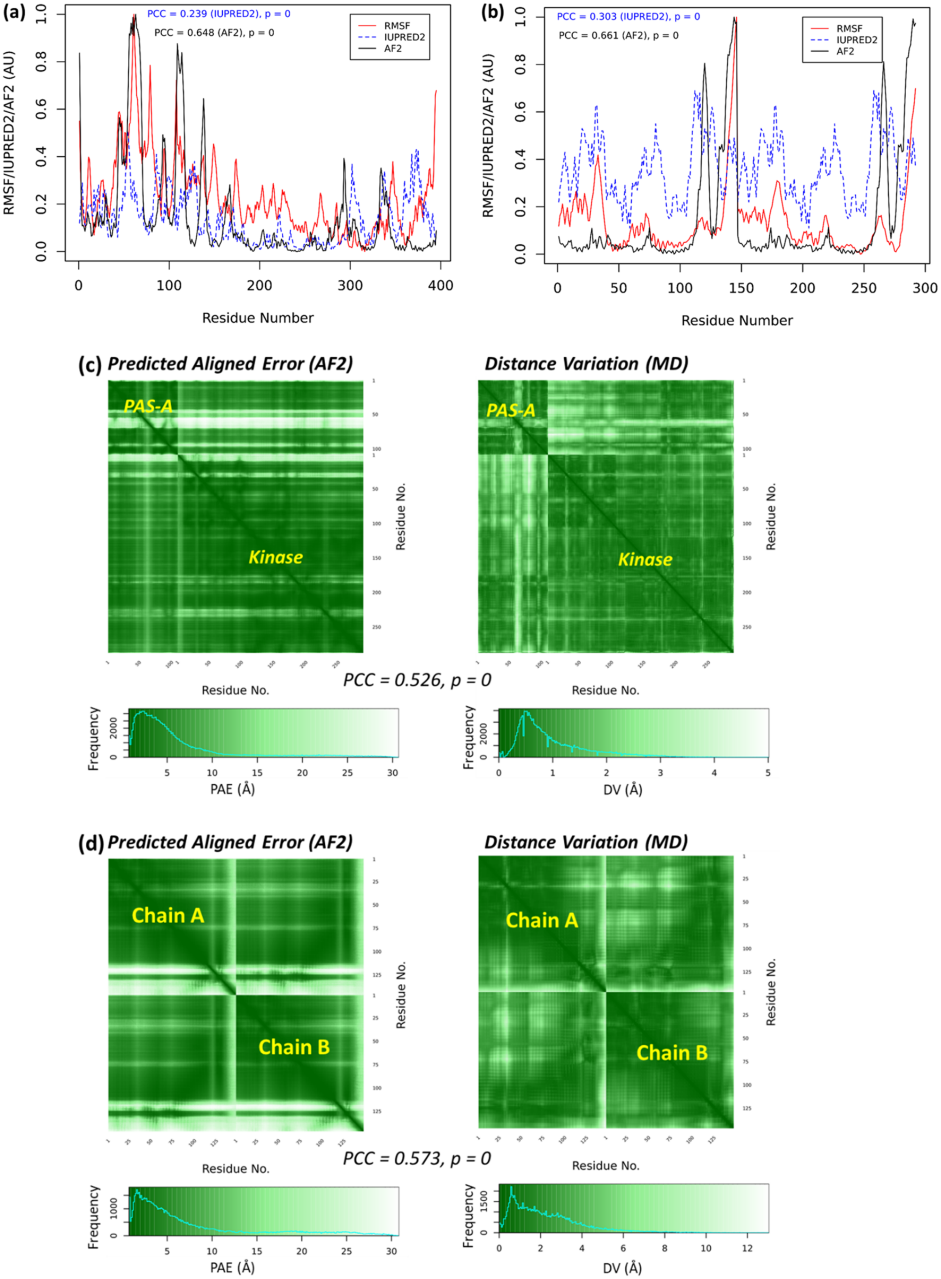
Multimers. The RMSF, the AF2 scores, and IUPRED2 scores of (**a**) the PAS-A and kinase heterodimer and (**b**) *M. tuberculosis* MerR homodimer. An arbitrary unit (AU) is used as both AF2 scores and RMSF are normalized.The PAE and DV maps of the PAS-A and kinase heterodimer (**c**) and *M. tuberculosis* MerR homodimer (**d**).

The modular protein–protein interaction network (PIN) in a living cell suggests that the protein functions are dependent on their interactions^68,69^. The AlphaFold-Multimer^8^ has been incorporated into AF2 to model the protein multimers一both homomers and heteromers一that is applicable to analyze the interactions and dynamics of the PPIs, at least in silico. RoseTTAFold^7^ was also applied with a similar approach to model cellular core complexes involved in key cellular functions such as transcription, translation and DNA repair^9^. The multimer models from AF2 or/and RoseTTAFold are extremely useful for understanding the PINs and modular protein functions. Known PINs are mainly based on curations of the experimental results such as those from the yeast two-hybrid experiments^70^. These networks are binary, i.e., the strength, or affinity, of the two interacting proteins are unknown^71^. The multimer models also provide the opportunity to simulate the protein–protein interaction strengths. Here, we modeled and simulated both a heterodimer and a homodimer to test the trends we observed in the monomers, as shown in Fig. 5.

In the heterodimer model, we used the sequences of the PAS-A domain and kinase domain as two independent entries, and applied AlphaFold-Multimer to construct the dimer structure. In this model, consistent with the monomer models, the AF2-scores correlate well with the RMSF from MD, indicating that the AlphaFold-Multimer also captures the residue flexibility (Fig. 5a). In addition, both the PAE and DV heatmaps (Fig. 5c) show interactions between the PAS-A and kinase “proteins”, consistent with the full PAS-kinase model. Therefore, beyond the multimer structures, AF2 can also predict the dynamics characteristics associated with the protein–protein interactions.

We used the sequence of a probable MerR family transcriptional regulatory protein from *Mycobacterium tuberculosis* (UniProt ID O53384). The monomer of this protein is available at the AF2 database at https://alphafold.ebi.ac.uk/entry/O53384. Using AlphaFold-multimer, the homodimer structure of this protein was constructed. Again, the AF2-score profile is consistent with the RMSF from MD (Fig. 5b), and the strong interactions between the two chains of this homodimer are shown in both the PAE and DV heatmaps (Fig. 5d), agreeing well with the known structures and dynamics of the Hg(II)-dependent MerR homodimer^59^. Moreover, the opening-and-closing dynamics of this homodimer was shown as the largest amplitude movement (PC1 from the principal component analysis of the MD trajectory), consistent with the previous simulations of the MerR family homodimers^59^.

### Intrinsically disordered and randomized proteins

Intrinsically disordered proteins (IDPs) or protein regions (IDPRs) are abundant in all organisms^72–74^. Proteins with structures deposited in the protein data bank^75^, even proteins with high-resolution X-ray structures, also contain significant disorder contents in their sequences^60,76^. It has been shown that the pLDDT scores provided by AF2 can also be applied to detect disorder^19^. For example, pLDDT scores lower than 50 are often indications of disorder in a protein^25,31^. The human structruome constructed by AF2 covers 58% of residues with a confident prediction (pLDDT > 70)^4^, indicating the prevalence of IDPs and IDPRs in proteomes^77^.

**Figure 6:**
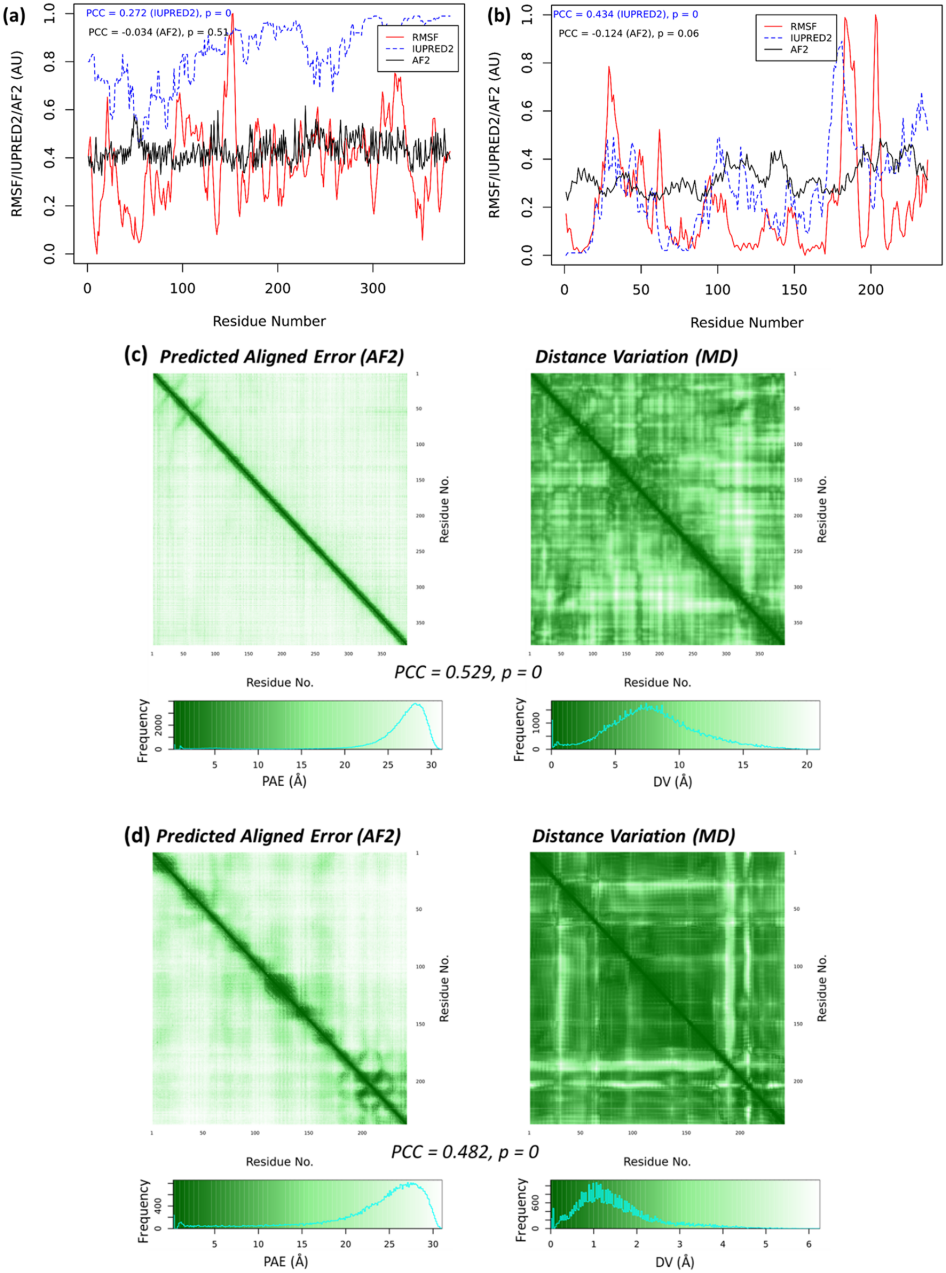
A fully disordered IDP, NVJP-1 (**a**, **c**) and a random protein (**b**, **d**). The RMSF (normalized), pLDDT/100 scores, and IUPRED2 scores for the IDP (**a**) and the random protein (**b**). The PAE and DV maps for the IDP (**c**) and random protein (**d**). Note: in 6 (**a**, **c**) the AF2-scores are replaced by pLDDT/100 for clarity.

We examined a fully disordered protein, NVJP-1 from the marine sandworm *Nereis virens*^78^. For this protein, no MSA hit has been found by AF2. The NVJP-1 protein is fully disordered, reflected by the IUPRED2 scores and the pLDDT scores (Fig. 6a). For clarity, the pLDDT scores are divided by 100 and are not normalized. Consistent with the IUPRED2 trend, all residues in NVJP-1 have a median pLDDT score of 42.8, and an IQR of 7.3, with 334 out of 387 residues demonstrating pLDDT scores lower than 50.0, suggesting disorder for these residues^31^. The RMSF profile of the NVJP-1 protein does not correlate with the AF2 (or pLDDT) scores, however, it shows a moderate correlation with the IUPRED scores (Fig. 6a). In addition, as indicated in the PAE and DV maps (Fig. 6c), all-atom superposition for the RMSF calculation is insufficient to estimate the flexibility of the residues due to large distance variations among the residues (also see Figs. 3A and S2).

We also compared the PAE and DV maps of NVJP-1 (Fig. 6c), which exhibited significant similarity (PCC = 0.529, *P* = 0). Unlike other globular proteins (Figs. 3c and S1 in the SI), the PAE or DV maps indicated that all residues in the protein have considerably high PAEs and DVs to other residues, even to their closely adjacent residues. The definition of “disordered” is as ambiguous as the definition of “ordered”^79^, given that rapid configurational dynamics in any protein continually occur in the cells^80^. Moreover, many IDPs may acquire folded structures upon interaction with a variety of partners, particularly, in the crowded cellular environment^81^. Here, we show that the AF2 models can be used to qualify the states of intrinsic disorder in proteins: large PAEs (and DVs) among adjacent residues serve as a signature of disorder.

We randomly constructed the amino acid sequence of a protein using the methods described previously^60^. For the randomized protein, AF2 did not find any MSA hits, similar to NVJP-1. However, unlike the NVJP-1 model that did not show any folded elements in its structure, the randomized protein contained folded regions (Fig. S1), indicating that fully disordered IDPs such as NVJP-1 do not occur by chance. This is in line with the previous results that randomized proteins have roughly half folded and half unfolded regions based on the disorder content calculations^60^. For both NVJP-1 and the randomized protein (Fig. 6a/b), the AF2-scores exhibit no or little correlation to the RMSF, indicating that AF2 cannot extract dynamics without co-evolutionary information from MSA (Table 1). As shown in Fig. 6d, the PAE/DV maps (PCC = 0.482, *P* = 0) of the random protein are featureless, and resemble those of NVJP-1. A recent study starts with the random “hallucination” sequences that also yields featureless 2D contact maps^15^. However, the contact maps can be optimized by interactions of Markov chain Monte Carlo simulations, and the optimized contact maps corresponded to highly featured protein folds validated by X-ray crystallography^15^. Therefore, the neural network used in AF2 and RoseTTAFold contains sufficiently rich structure and dynamics information for useful protein engineering.

### Other models, protein disorder, residue flexibility and AF2 scores

**Figure 7:**
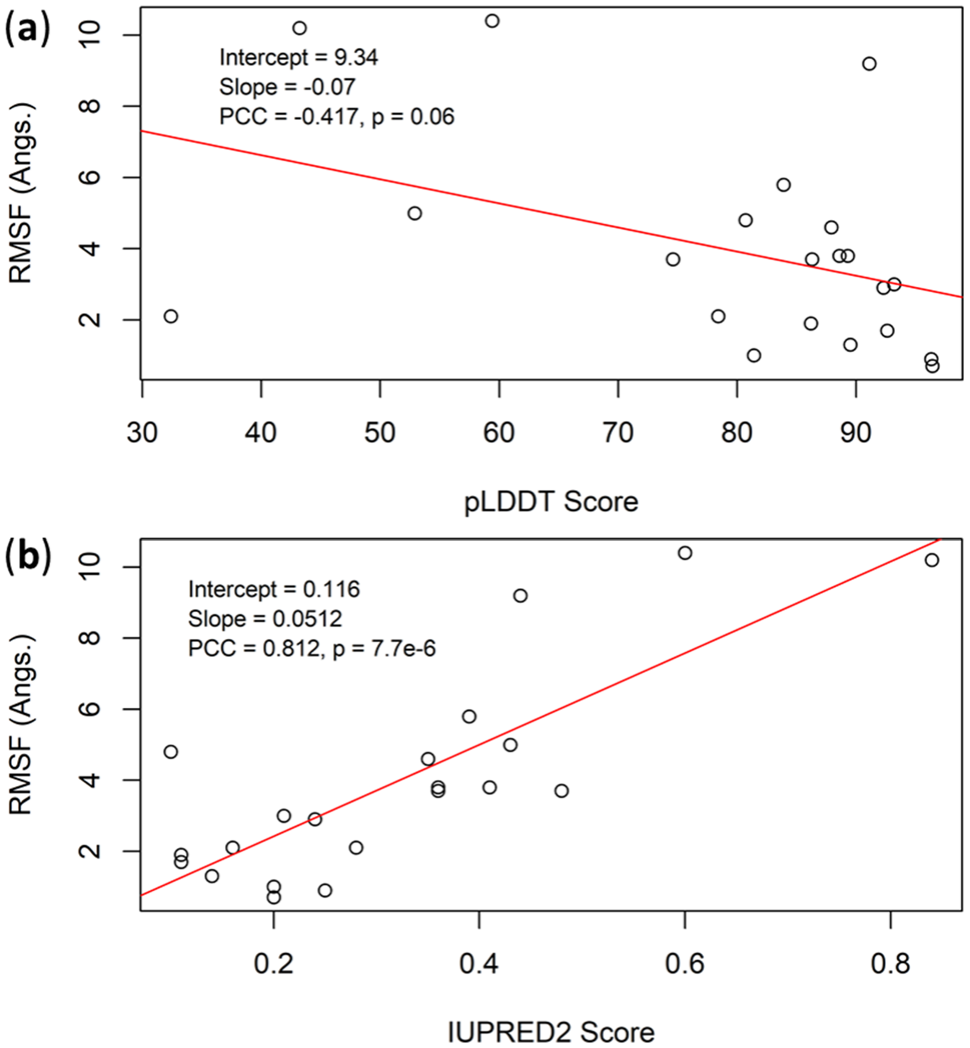
Linear regression between (**a**) RMSF and pLDDT, (**b**) RMSF and IUPRED2 based on 21 protein models from Tables 1 and S1.

Besides the 11 models discussed above, we analyzed 10 more models from the budding yeast (*Saccharomyces cerevisiae*), as listed in Table S1 and Fig. S6. To select these proteins, first, all yeast proteins (ca. 6000) were classified 10 groups based on the quantiles of the Hirsch-index (H-index) centrality of the protein–protein interaction network (PIN, adopted from Ref.^71^). The PINs, similar to other natural networks, have power-law distributions of the node degrees^82^. The H-index^83^ (originally proposed for quantifying the research performances of researchers) centrality is a measure that connect both node degree and coreness^84^. Using these models, together with the models shown in Table 1 and Fig. 1, we calculated the overall agreement between the residue flexibility (RMSF from MD) and the per-residue pLDDT scores and the IUPRED2 disorder contents, as shown in Fig. 7. A strong correlation between the mean IUPRED2 scores and mean RMSF is observed (Fig. 7b), but that between mean pLDDT and mean RMSF is less significant (Fig. 7b). However, the per-residue pLDDT scores are highly correlated to the per-residue RMSF (e.g., Figs. 2, 3, 4, 5, 6), yet the IUPRED scores do not correlate to the RMSF and in certain cases even contradict the RMSF scores, for example, models c/d (Fig. 2c/2d) and model g (Fig. 4b) in the main text, and models for the yeast proteins MRPL20, ALG5 and VTI1 in the SI.

Protein disorder is ubiquitous. The pLDDT scores have been considered as a predictor for the residue disorder contents: the residues with the pLDDT scores lower than 50 may be located in the IDPR^19^. The aim of the present work is to add an additional time dimension to the 3D protein structures, i.e., to explore the protein dynamics, which can also be understood from the protein residue flexibilities. Intrinsic protein disorder is strongly related to protein flexibilities^55^. It was suggested that it is the protein flexibility that should carry the term “intrinsic”, but not the disorder^85^. Here, we show that in all models listed in Table 1 the AF2 scores (or the reversed pLDDT scores) significantly correlate with the RMSF scores obtained from MD simulations.

The success of AF2 is derived from translating the evolutionary knowledge gained from MSA to distance contact matrices. The ensembles of sequences, in principle, also represent the ensembles of structures, among which the structural variations tend to aggregate toward the evolutionarily stable states. Therefore, the multisequence alignment or multistructure alignment should propagate along similar trajectories, which had been verified in both principal component analysis^86^ and the elastic network models^87^. It is, therefore, possible to decode the dynamics information encoded in the protein sequences from the evolutionary history, i.e., MSA.

## Conclusions

We show here that the structural models predicted by AlphaFold2 not only produce the atomic coordinates (or 3D fold) of the proteins, but also give important information regarding residue flexibility associated with the protein dynamics, which are comparable to the results from molecular dynamics simulations. For globular proteins and protein complexes, the AF2-scores derived from the pLDDT scores (i.e., the confidence scores evaluated by AF2) are highly consistent with the RMSF profiles from MD. For these protein models, the PAE maps predicted by AF2 also showed consistent trends as the distance variation maps from MD. Anfinsen's rule illustrates that the protein structure is determined by its primary sequence. This rule also indicates that the protein structure determines its function, which is derived from protein dynamics. Our results suggest that scores from the AF2 models are able to infer protein dynamics, which is crucial to understand protein interactions and functions. The low pLDDT scores in the AF2 models might not result from “low confidence” but be attributed to high residue flexibility, which contributes to protein function.

## Supplementary Information

Supplementary Information.

## Data Availability

All data generated or analyzed during this study are included in this published article and its supplementary information files.
