# Supplementary material for: AlphaFold2 models indicate that protein sequence determines both structure and dynamics
Source: Sci Rep. 2022 Jun 23;12:10696. doi: 10.1038/s41598-022-14382-9 (PMC9226352; doi:10.1038/s41598-022-14382-9)
Supplement: Supplementary file 1 — Supplementary Information. [file 41598_2022_14382_MOESM1_ESM.docx]

Supplementary Information for

AlphaFold2 models indicate that protein sequence determines both structure and dynamics

Hao-Bo Guo^1,2^, Alexander Perminov^1,3^, Selemon Bekele^1,2^, Gary Kedziora^4^, Sanaz Farajollahi^1,2^, Vanessa Varaljay^1^, Kevin Hinkle^5^, Valeria Molinero^6^, Konrad Meister^7,8^, Chia Hung^1^, Patrick Dennis^1^, Nancy Kelley-Loughnane^1*^, Rajiv Berry^1*^

1. Materials and Manufacturing Directorate, Air Force Research Laboratory, WPAFB, OH

2. UES Inc., Dayton, OH

3. Computer Science Department, Miami University, Oxford, OH

4. General Dynamics Information Technology, Inc., WPAFB, OH

5. Department of Chemical and Materials Engineering, Dayton University, Dayton, OH

6. Department of Chemistry, The University of Utah, Salt Lake City, UT

7. Department of Natural Sciences, University of Alaska Southeast, Juneau, AK

8. Max Planck Institute for Polymer Research, Mainz, Germany

*Correspondence: nancy.kelley-loughnane.1@us.af.mil (NKL) and rajiv.berry@us.af.mil (RB)

The Supplementary Information Includes:

**Figure S1**. Structures of the models used in present work.

**Figure S2**. The RMSF values from MD simulation of the GNE protein, calculated from all-atom superposition.

**Figure S3**. The RMSF values from MD simulation of the LanM protein, calculated from a domain-specific superposition.

**Figure S4**. A hidden symmetry from inter-domain dynamics.

**Figure S5**. PAE versus DV heatmaps.

**Figure S6**. Structures of the 10 yeast (*S. cerevisiae*) models.

**Table S1**. Additional proteins models from *S. cerevisiae.*

***Appendix***: the sequences of the proteins


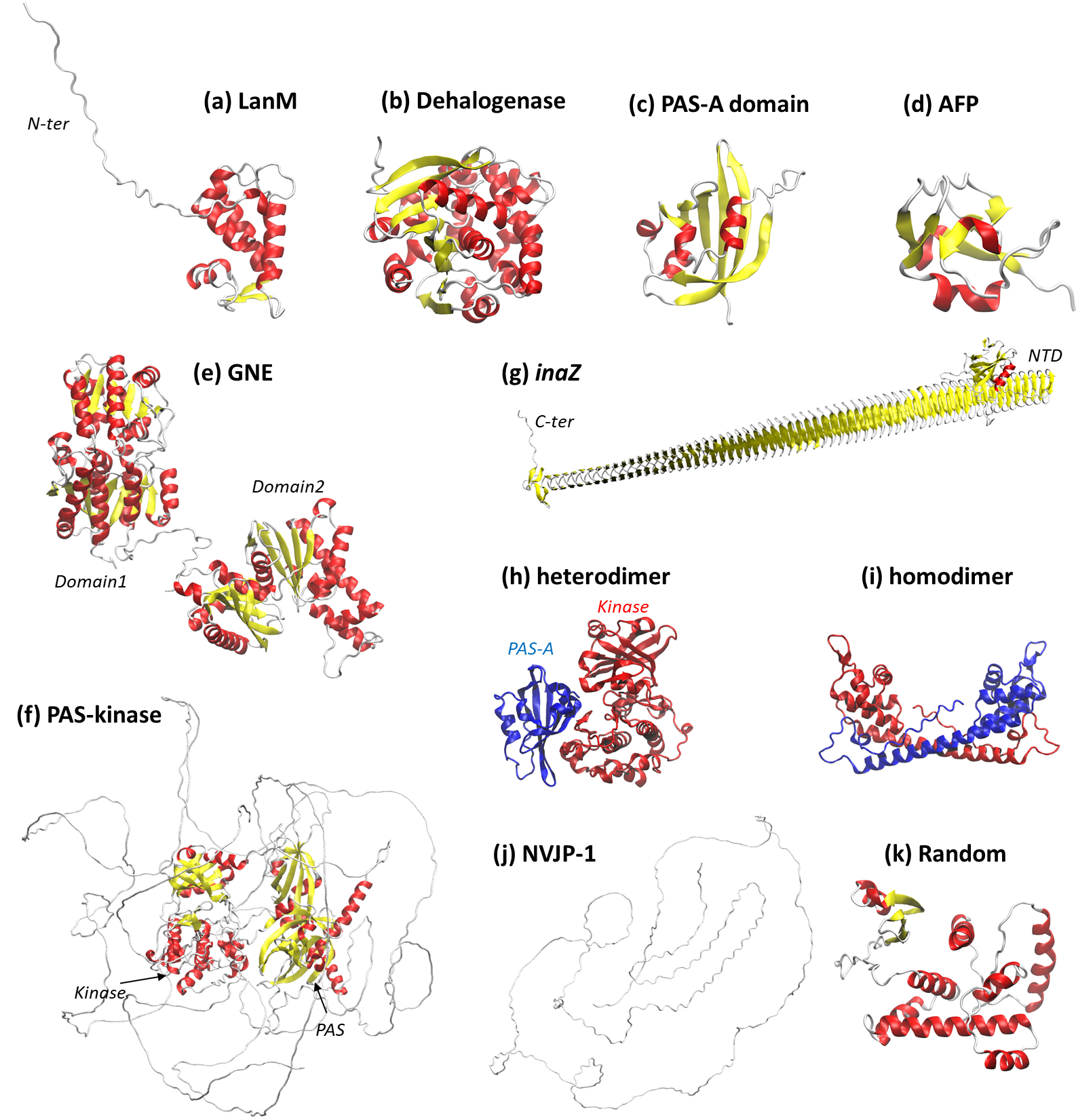


**Figure S1**. Structures of the models used in present work, including (a) full length LanM (with the N-terminus labeled), (b) a dehalogenase, (c) the PAS-A domain protein, (d) an antifreeze protein, (e) the two domain protein GNE, (f) the PAS-kinase with both the PAS-domain and the kinase domain labeled, (g) the *inaZ* protein wi the the N-terminal domain (NTD) and disordered C-terminus labeled, (h) a heterodimer with PAS-A domain protein and the kinase, (i) a homodimer of MtMerR, (j) the fully disordered protein NVJP-1, (k) a randomized protein. In the multimer models (h and i), the two chains are colored in blue and red respectively. In all other models, the helical regions (ɑ-helix and 3_10_-helix) are colored in red, the β-sheets are colored in yellow, and the coil regions (including turns, bridges) are colored in gray.


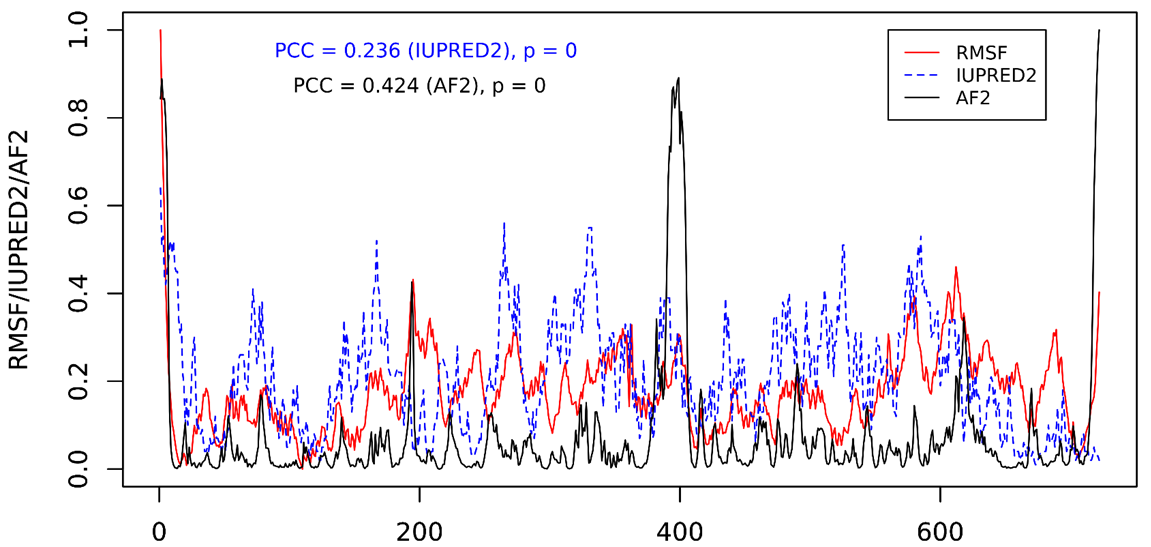


**Figure S2**. The RMSF values from MD simulation of the GNE protein, calculated from all-atom superposition. The AF2-scores are less correlated to the RMSF, compared to the RMSF calculated from a domain-specific approach (Figure 3a). The all-atom superposition indicates a more rigid linker region (residues 381 to 401), contradicting the principal component analysis.


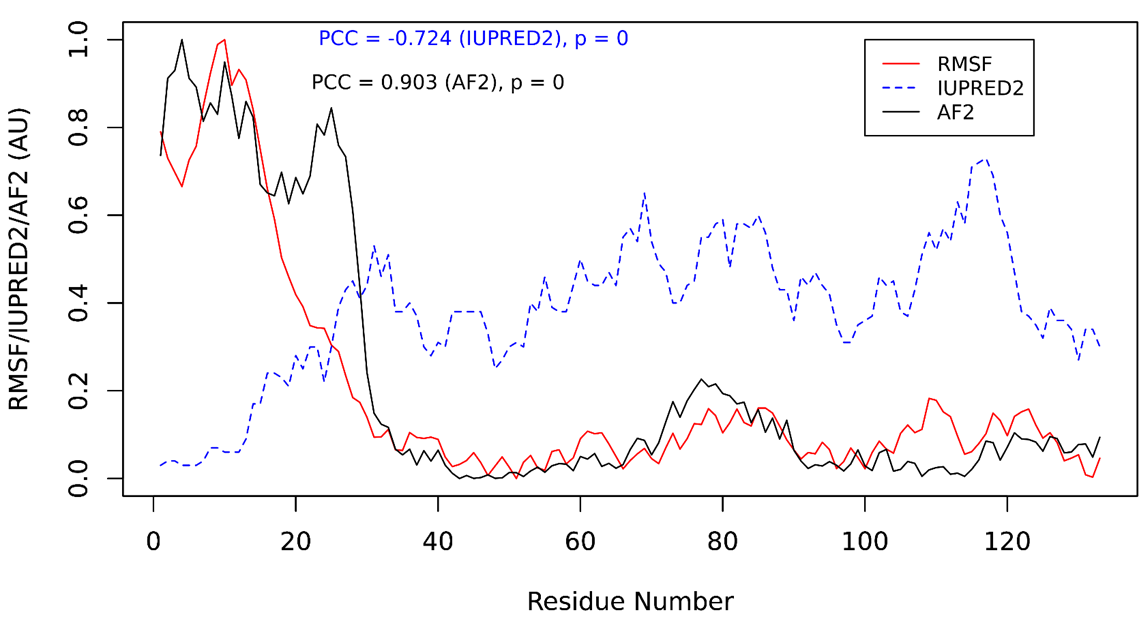


**Figure S3**. The RMSF values from MD simulation of the LanM protein, calculated from a domain-specific superposition. The AF2 scores are more strongly correlated to the RMSF calculated compared to an all-atom superposition approach (Figure 2a).


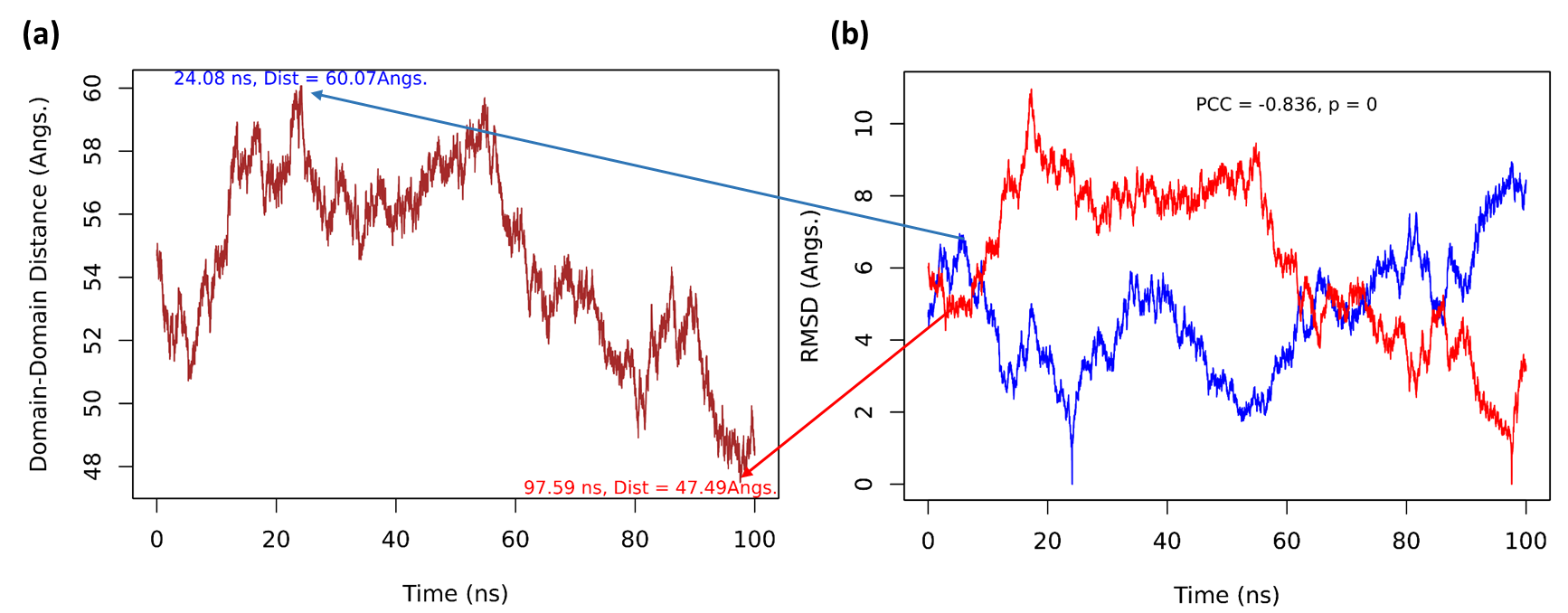


**Figure S4**. A hidden symmetry from inter-domain dynamics.(a) During the 100 ns MD simulation, the center-of-mass (COM) distance between the two domains of the GNE protein varies from 47.5 to 60 Å. (b) The RMSD profiles were monitored using the configurations with two extreme COM values, either to the maximal (red, at 24.08 ns, COM = 60.07 Å) or the minimal (blue, at 97.59 ns, COM = 47.49 Å) show a mirror symmetry: both RMSD profiles are anticorrelated (PCC = -0.836, p = 0).


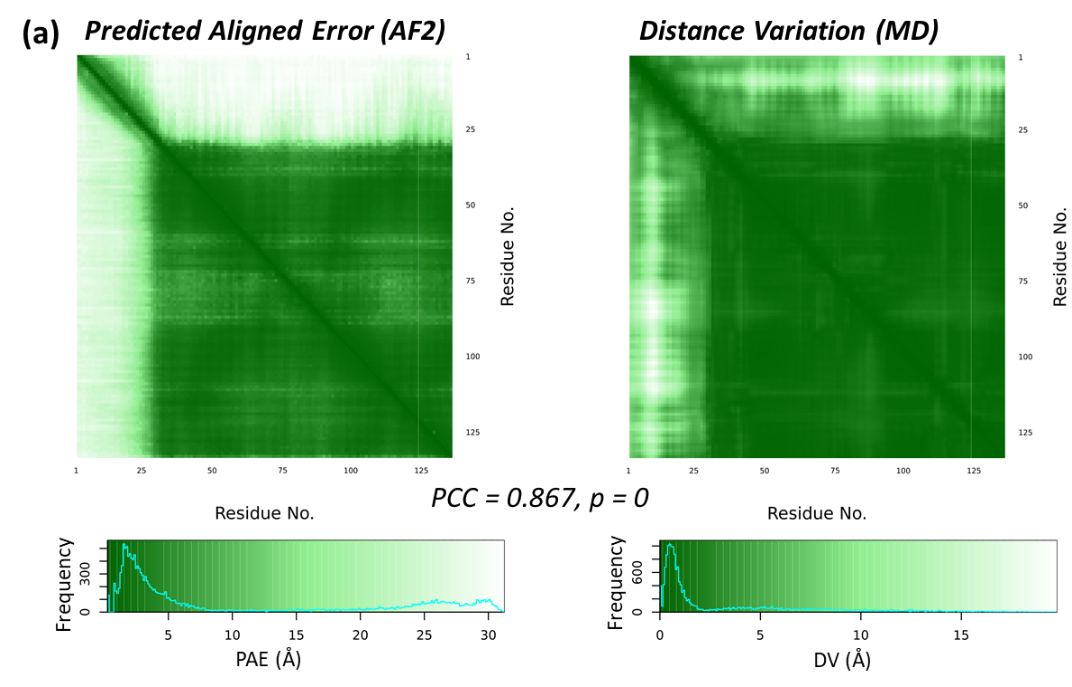


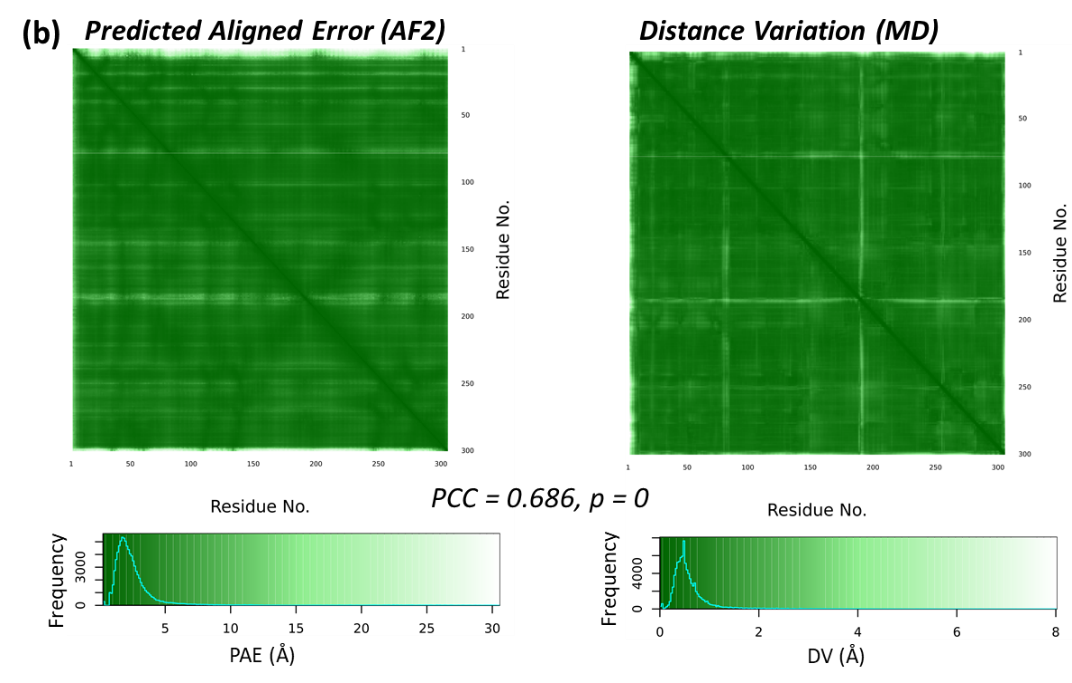


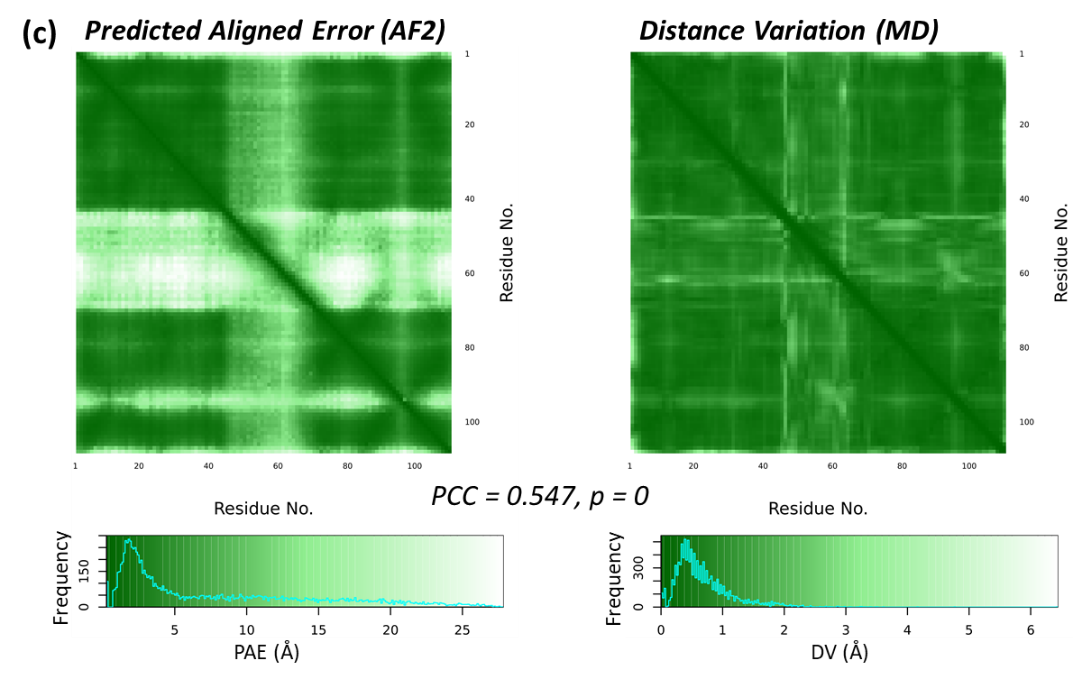


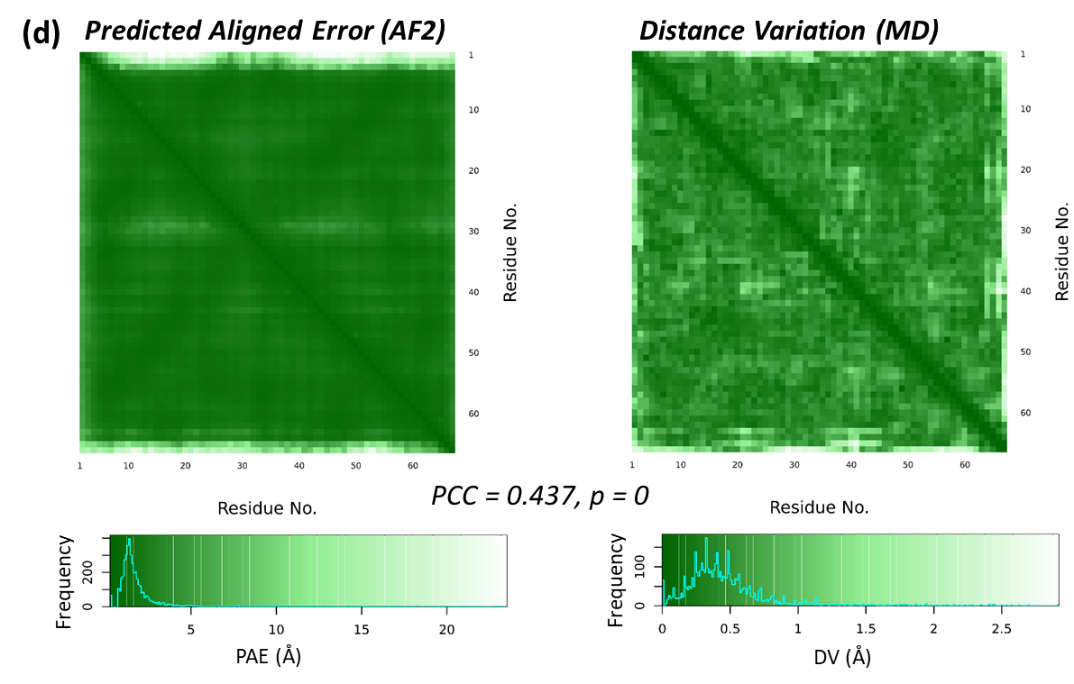


**Figure S5**. PAE versus DV heatmaps for the protein systems shown in Figure 2 (main text). (a) LanM; (b) dehalogenase; (c) PAS-domain protein; (d) antifreeze protein (AFP). The flexible N-terminus of LanM (a) can be clearly seen in both the PAE and DV maps. Both the dehalogenase and AFP are well-folded globular proteins and the PAE and DV have quasi-Gaussian distributions (see histograms in the color bars in b and d). The PAS-domain protein (c) has a relatively flexible linker in the middle (residues 47 to 70) as shown in Figure 2c, rendering a relatively low correlation between the AF2 scores and RMSF, compared to the other three models. Correlation data between the PAE and DV matrices are provided in the Figures.


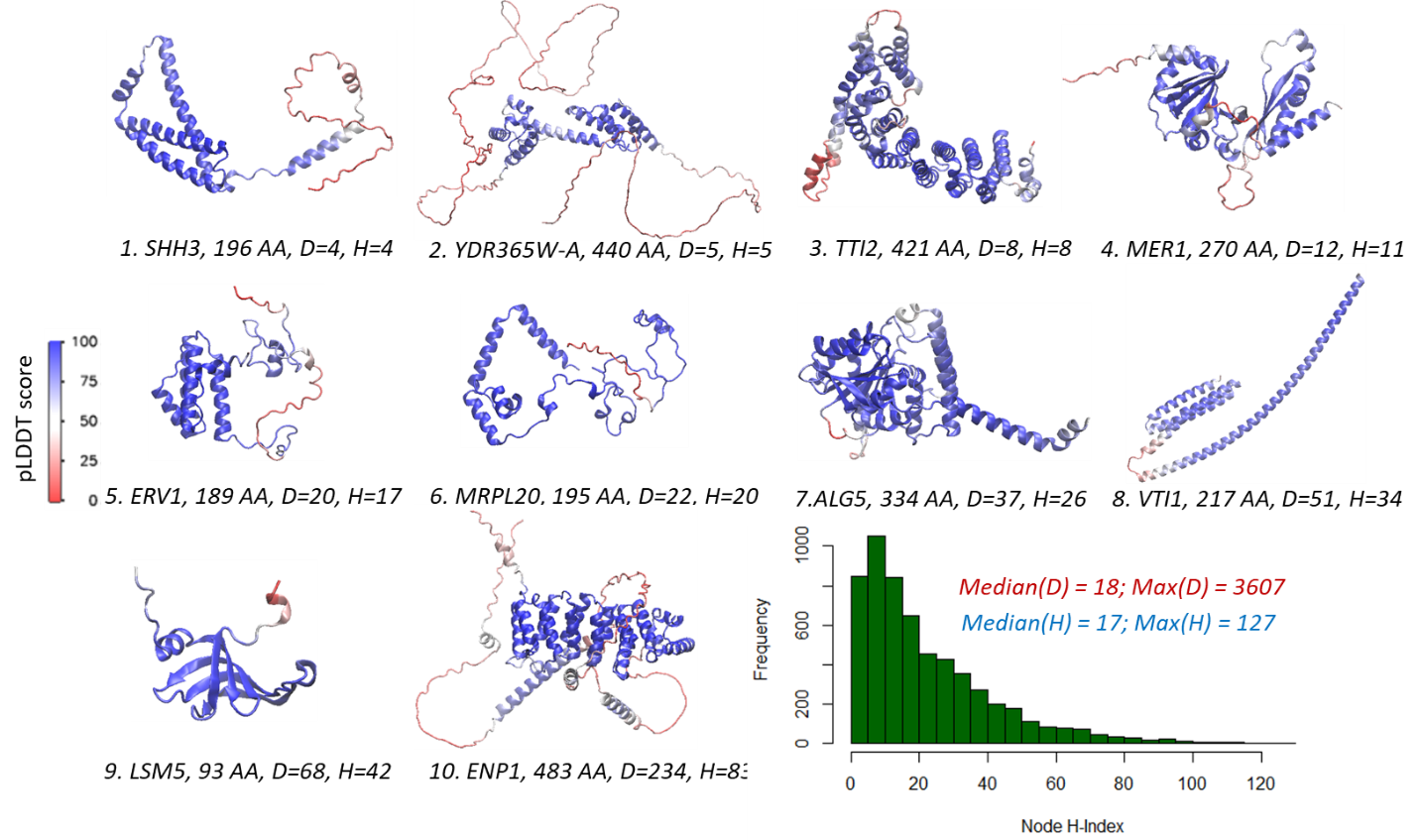


**Figure S6**. Structures of the 10 yeast (*S. cerevisiae*) models randomly selected from each of the 10 quantiles of the H-index centrality of the protein-protein interaction network. The inset (right bottom) is a histogram of the node H-index: for a protein that interacts with N proteins (including itself), each of these N proteins also interact with at least N proteins, the maximal value of N is defined as the H-index. All proteins are colored by the per-residue pLDDT scores with the color bar shown at left.

**Table S1**. Additional proteins models from *S. cerevisiae* and based on the yeast protein-protein interaction network.

| **Protein** | **AA^1^** | **H-index^2^** | **MSA^3^** | **pLDDT^4^** | **IUPRED2^4^** | **RMSF (Å)^4^** | **PCC** | **Slope** | **Int.** |
| --- | --- | --- | --- | --- | --- | --- | --- | --- | --- |
| SSH3 | 196 | 4 | 1139 | 80.7±21.9 | 0.10±0.11 | 4.8±2.6 | -0.66 | -5.6 | 107 |
| YDR365W-A | 440 | 5 | 2031 | 59.4±25.2 | 0.60±0.28 | 10.4±5.3 | -0.61 | -2.9 | 89 |
| TTI2 | 421 | 8 | 1354 | 86.2±14.0 | 0.11±0.11 | 1.9±1.3 | -0.85 | -9.5 | 104 |
| MER1 | 270 | 11 | 1722 | 78.4±19.1 | 0.16±0.16 | 2.1±1.4 | -0.75 | -10.0 | 99 |
| ERV1 | 189 | 17 | 1979 | 86.3±19.8 | 0.48±0.16 | 3.7±2.1 | -0.68 | -6.5 | 111 |
| MRPL20 | 195 | 20 | 1741 | 91.1±16.4 | 0.44±0.12 | 9.2±3.1 | -0.27 | -1.4 | 104 |
| ALG5 | 334 | 26 | 3775 | 92.6±8.0 | 0.11±0.12 | 1.7±1.7 | -0.35 | -1.6 | 95 |
| VTI1 | 217 | 34 | 1397 | 87.9±10.6 | 0.35±0.19 | 4.6±1.9 | -0.26 | -1.5 | 95 |
| 9. LSM5 | 93 | 42 | 1110 | 92.3±11.2 | 0.24±0.17 | 2.9±2.7 | -0.78 | -3.2 | 102 |
| 10. ENP1 | 483 | 83 | 1978 | 74.6±25.6 | 0.36±0.28 | 3.7±2.7 | -0.64 | -6.2 | 97 |

1. Number of amino acid residues.

2. The H-index centrality from the protein-protein interaction network. All proteins are randomly selected from 10 evenly distributed quantile sets of the H-indices.

3. The MSA hits from the BFD (Big Fantastic Database). The MSA hits include those that match the protein partial segments.

4. Mean ± SD for per-residue pLDDT, IUPRED2 and RMSF values.

5. The Pearson’s correlation coefficient (PCC) between pLDDT and RMSF scores, the slope and intercepts of the linear fitting between them are also listed; note that as pLDDT and the AF2 scores in this work are anticorrelated.

***Appendix***: The sequences of the proteins studied in the present work

Part 1. Proteins shown in Table 1 and Figure S1.

>a. LanM

MAFRLSSAVLLAALVAAPAYAAPTTTTKVDIAAFDPDKDGTIDLKEALAAGSAAFDKLDPDKDGTLDAKELKGRVSEADLKKLDPDNDGTLDKKEYLAAVEAQFKAANPDNDGTIDARELASPAGSALVNLIR

>b. Dehalogenase from *Delftia acidovorans*

MHTDPWMPGLRQQRITVDDGVEINAWVGGQGPALLLVHGHPQTSAIWHRVAPRLAQQFTVVLADLRGYGDSSRPAGDPEHVNYSKRTMARDLLRLMARLGHEHFSVLAHDRGARVAHRLAMDYPASVQRLVLLDIAPTLAMYEQTGEAFARAYWHWFFLIQPAPLPERLIEADPAAYVREIMGRRSAGLAPFDPRALAEYQRCLALPGSAHGMCEDYRASAGIDLDHDREDRQLGRRLSMPLLVLWGEEGVVHRCFDPLREWQLVADDVRGRPLACGHYIAEEAPDALLDAALPFLLQAG

>c. PAS-A domain protein

MNKAIFTVDAKTTEILVANDKACGLLGYSSQDLIGQKLTQFFLRSDSDVVEALSEEHMEADGHAAVVFGTVVDIISRSGEKIPVSVWMKRMRQERRLCCVVVLEPVER

>d. Antifreeze protein (AFP)

MNQASVVANQLIPINTALTLVMMRSEVVTPVGIPAEDIPRLVSMQVNRAVPLGTTLMPDMVKGYAA

>e. GNE (two domain protein)

MEKNGNNRKLRVCVATCNRADYSKLAPIMFGIKTEPEFFELDVVVLGSHLIDDYGNTYRMIEQDDFDINTRLHTIVRGEDEAAMVESVGLALVKLPDVLNRLKPDIMIVHGDRFDALALATSAALMNIRILHIEGGEVSGTIDDSIRHAITKLAHYHVCCTRSAEQHLISMCEDHDRILLAGCPSYDKLLSAKNKDYMSIIRMWLGDDVKSKDYIVALQHPVTTDIKHSIKMFELTLDALISFNKRTLVLFPNIDAGSKEMVRVMRKKGIEHHPNFRAVKHVPFDQFIQLVAHAGCMIGNSSCGVREVGAFGTPVINLGTRQIGRETGENVLHVRDADTQDKILQALHLQFGKQYPCSKIYGDGNAVPRILKFLKSIDLQEPLQKKFCFPPVKENISQDIDHILETLSALAVDLGGTNLRVAIVSMKGEIVKKYTQFNPKTYEERINLILQMCVEAAAEAVKLNCRILGVGISTGGRVNPREGIVLHSTKLIQEWNSVDLRTPLSDTLHLPVWVDNDGNCAALAERKFGQGKGLENFVTLITGTGIGGGIIHQHELIHGSSFCAAELGHLVVSLDGPDCSCGSHGCIEAYASGMALQREAKKLHDEDLLLVEGMSVPKDEAVGALHLIQAAKLGNAKAQSILRTAGTALGLGVVNILHTMNPSLVILSGVLASHYIHIVKDVIRQQALSSVQDVDVVVSDLVDPALLGAASMVLDYTTRRIY

>f. PAS-A containing kinase

MEDGGLTAFEEDQRCLSQSLPLPVSAEGPAAQTTAEPSRSFSSAHRHLSRRNGLSRLCQSRTALSEDRWSSYCLSSLAAQNICTSKLHCPAAPEHTDPSEPRGSVSCCSLLRGLSSGWSSPLLPAPVCNPNKAIFTVDAKTTEILVANDKACGLLGYSSQDLIGQKLTQFFLRSDSDVVEALSEEHMEADGHAAVVFGTVVDIISRSGEKIPVSVWMKRMRQERRLCCVVVLEPVERVSTWVAFQSDGTVTSCDSLFAHLHGYVSGEDVAGQHITDLIPSVQLPPSGQHIPKNLKIQRSVGRARDGTTFPLSLKLKSQPSSEEATTGEAAPVSGYRASVWVFCTISGLITLLPDGTIHGINHSFALTLFGYGKTELLGKNITFLIPGFYSYMDLAYNSSLQLPDLASCLDVGNESGCGERTLDPWQGQDPAEGGQDPRINVVLAGGHVVPRDEIRKLMESQDIFTGTQTELIAGGQLLSCLSPQPAPGVDNVPEGSLPVHGEQALPKDQQITALGREEPVAIESPGQDLLGESRSEPVDVKPFASCEDSEAPVPAEDGGSDAGMCGLCQKAQLERMGVSGPSGSDLWAGAAVAKPQAKGQLAGGSLLMHCPCYGSEWGLWWRSQDLAPSPSGMAGLSFGTPTLDEPWLGVENDREELQTCLIKEQLSQLSLAGALDVPHAELVPTECQAVTAPVSSCDLGGRDLCGGCTGSSSACYALATDLPGGLEAVEAQEVDVNSFSWNLKELFFSDQTDQTSSNCSCATSELRETPSSLAVGSDPDVGSLQEQGSCVLDDRELLLLTGTCVDLGQGRRFRESCVGHDPTEPLEVCLVSSEHYAASDRESPGHVPSTLDAGPEDTCPSAEEPRLNVQVTSTPVIVMRGAAGLQREIQEGAYSGSCYHRDGLRLSIQFEVRRVELQGPTPLFCCWLVKDLLHSQRDSAARTRLFLASLPGSTHSTAAELTGPSLVEVLRARPWFEEPPKAVELEGLAACEGEYSQKYSTMSPLGSGAFGFVWTAVDKEKNKEVVVKFIKKEKVLEDCWIEDPKLGKVTLEIAILSRVEHANIIKVLDIFENQGFFQLVMEKHGSGLDLFAFIDRHPRLDEPLASYIFRQLVSAVGYLRLKDIIHRDIKDENIVIAEDFTIKLIDFGSAAYLERGKLFYTFCGTIEYCAPEVLMGNPYRGPELEMWSLGVTLYTLVFEENPFCELEETVEAAIHPPYLVSKELMSLVSGLLQPVPERRTTLEKLVTDPWVTQPVNLADYTWEEVFRVNKPESGVLSAASLEMGNRSLSDVAQAQELCGGPVPGEAPNGQGCLHPGDPRLLTS

>g. inaZ

MNLDKALVLRTCANNMADHCGLIWPASGTVESRYWQSTRRHENGLVGLLWGAGTSAFLSVHADARWIVCEVAVADIISLEEPGMVKFPRAEVVHVGDRISASHFISARQADPASTSTSTLTPMPTAIPTPMPAVASVTLPVAEQARHEVFDVASVSAAAAPVNTLPVTTPQNVQTATYGSTLSGDNHSRLIAGYGSNETAGNHSDLIAGYGSTGTAGSDSWLVAGYGSTQTAGGDSALTAGYGSTQTAREGSNLTAGYGSTGTAGSDSSLIAGYGSTQTSGGDSSLTAGYGSTQTAQEGSNLTAGYGSTGTAGSDSSLIAGYGSTQTSGGDSSLTAGYGSTQTAQEGSNLTAGYGSTGTAGVDSSLIAGYGSTQTSGSDSALTAGYGSTQTAQEGSNLTAGYGSTGTAGSDSSLIAGYGSTQTSGSDSSLTAGYGSTQTAQEGSILTAGYGSTGTAGVDSSLIAGYGSTQTSGSDSALTAGYGSTQTAQEGSNLTAGYGSTGTAGADSSLIAGYGSTQTSGSESSLTAGYGSTQTAREGSTLTAGYGSTGTAGADSSLIAGYGSTQTSGSESSLTAGYGSTQTAQQGSVLTSGYGSTQTAGAASNLTTGYGSTGTAGHESFIIAGYGSTQTAGHKSILTAGYGSTQTARDGSDLIAGYGSTGTAGSGSSLIAGYGSTQTASYRSMLTAGYGSTQTAREHSDLVTGYGSTSTAGSNSSLIAGYGSTQTAGFKSILTAGYGSTQTAQERTSLVAGYGSTSTAGYSSSLIAGYGSTQTAGYESTLTAGYGSTQTAQENSSLTTGYGSTSTAGYSSSLIAGYGSTQTAGYESTLTAGYGSTQTAQERSDLVTGYGSTSTAGYASSLIAGYGSTQTAGYESTLTAGYGSTQTAQENSSLTTGYGSTSTAGFASSLISGYGSTQTAGYKSTLTAGYGSTQTAEYGSSLTAGYGSTATAGQDSSLIAGYGSSLTSGIRSFLTAGYGSTLIAGLRSVLIAGYGSSLTSGVRSTLTAGYGSNQIASYGSSLIAGHESIQVAGNKSMLIAGKGSSQTAGFRSTLIAGAGSVQLAGDRSRLIAGADSNQTAGDRSKLLAGNNSYLTAGDRSKLTGGHDCTLMAGDQSRLTAGKNSVLTAGARSKLIGSEGSTLSAGEDSILIFRLWDGKRYRQLVARTGENGVEADIPYYVNEDDDIVDKPDEDDDWIEVK

>h. PAS-A domain and kinase domain heterodimer

>h1. PAS-A domain | chain A

MNKAIFTVDAKTTEILVANDKACGLLGYSSQDLIGQKLTQFFLRSDSDVVEALSEEHMEADGHAAVVFGTVVDIISRSGEKIPVSVWMKRMRQERRLCCVVVLEPVER

>h2. Kinase domain | chain B

MKAVELEGLAACEGEYSQKYSTMSPLGSGAFGFVWTAVDKEKNKEVVVKFIKKEKVLEDCWIEDPKLGKVTLEIAILSRVEHANIIKVLDIFENQGFFQLVMEKHGSGLDLFAFIDRHPRLDEPLASYIFRQLVSAVGYLRLKDIIHRDIKDENIVIAEDFTIKLIDFGSAAYLERGKLFYTFCGTIEYCAPEVLMGNPYRGPELEMWSLGVTLYTLVFEENPFCELEETVEAAIHPPYLVSKELMSLVSGLLQPVPERRTTLEKLVTDPWVTQPVNLADYTWEEVF

>i. MtMerR homodimer

>i1. MtMerR homodimer | chain A

MKISEVAALTNTSTKTLRFYENSGLLPPPARTASGYRNYGPEIVDRLRFIHRGQAAGLALQEVRQILAIHDRGEAPCAHVRQLLSTRIDEVRAQIAELIALEGHLQTLLDHASYGPPTEHDHSTVCWILESDLDEPTAIEVSDIHA

>i2. MtMerR homodimer | chain B

MKISEVAALTNTSTKTLRFYENSGLLPPPARTASGYRNYGPEIVDRLRFIHRGQAAGLALQEVRQILAIHDRGEAPCAHVRQLLSTRIDEVRAQIAELIALEGHLQTLLDHASYGPPTEHDHSTVCWILESDLDEPTAIEVSDIHA

>j. NVJP-1 (IDP)

HNDGYGHDDHHGHGHGGYGGHGHGDYGGHGHGGYGGHGHGHGHGHGHFDDHPFYTIPAFGHGYGHGHGGHGHGYGGHDGYGGHGGYGGHGGYGDHGHGGHGYGSHGGHGQDYGGDYGGHGHGGHHHGGHDHDDFGHDFGHHGGDHGHHGGGHHGHHDGYGHDQGHGHGHGDYDYSHGNQHDNGHREHYGGHQAAGHHGHGESGSHYGGQGGGGGSHHSGGHDGSHNHGKFQTQYSYRGNDKYGGHNQYQGHGAYNEYSKSKGTGKYVAHGTEEGHHQLDGYEKDNHQGKYEGHGEHHSHQHGDGHHKRKEHGDHAGISGFHGQGHVQGNHALHGHHGGGHGHHGGEHGHSARGHHGGGGYGGGGHGHRGHHGGHQGHHGHH

>k. Randomized protein

YANLLWPWVFACTFAYMNHEYSSLQCNTQDIMMEDRDHYNNDIFPAIKGTNQGCGHWFCMGADYHESFPIILMLSHQKRLWSKLLMSSYLCVDQTEQEHMADFFEDEETRLFSMANHAHRIAMTQMCGMYRNYWSHEDFRHYMFQPDNVFHHATCHGLHVFVRILNLGCSTPEAPICVERTDQDPRGAEQDFVCYCMILCNGDERADHIFCKSQVFPAIAPIPCKDQKSRSFMTACG

Part 2. Proteins shown in Table S1 and Figure S6.

>YMR118C|SHH3

MKATIQRVTSVFGVPRASVFVPRISTPFILHNYISNGRMDLFSKEFHNGRVSKSDLWSSNKEEELLVSQRKKRPISPHLTVYEPEMSWYLSSLHRISGVLLALGFYAFTITLGVTTIMGMDTTFQDLNKWYHEKMPKWSQWVAKGSAAYLFAFHFGNGIRHLIWDMGYELTNRGVIKTGSIVLAGTLVLGTYLLAQ

>YDR365W-A|YDR365W-A

MESQQLSQHSPISHGSACASVTSKEVHTNQDPLDVSASKTEECEKASTKANSQQTTTPASSAVPENPHHASPQPASVPPPQNGPYPQQCMMTQNQANPSGWSFYGHPSMIPYTPYQMSPMYFPPGPQSQFPQYPSSVGTPLSTPSPESGNTFTDSSSADSDMTSTKKYVRPPPMLTSPNDFPNWVKTYIKFLQNSNLGGIIPTVNGKPVRQITDDELTFLYNTFQIFAPSQFLPTWVKDILSVDYTDIMKILSKSIEKMQSDTQEANDIVTLANLQYNGSTPADAFETKVTNIIDRLNNNGIHINNKVACQLIMRGLSGEYKFLRYTRHRHLNMTVAELFLDIHAIYEEQQGSRNSKPNYRRNLSDEKNDSRSYTNTTKPKVIARNPQKTNNSKSKTARAHNVSTSNNSPSTDNDSISKSTTEPIQLNNKHDLHLRPGTY

>YJR136C|TTI2 MTAVTDIIDELNDSSLSSTRLRELCLQLRKKTDTGCAITVSDEVNLIESLSYHSISPGVDIQINTDVLQTIDYYFQRNKSEHDEIMCVLISKLQPLLLKRKSNFELKEQRNLGLKPTLGMSLKEDNLMQAWVSQGGLKGIPLFYVILLHLKRRDISTNLSWIIPGILNILDDTTDIRRIKLRGVLLLQTLLNHTFMNETNDSKWIQFSSTGLFPLFEKTLINMCYFLPPSYNADETIAIWRVVFPTIQSLYKVEFLDNYTKYQYHLEKFMSEIILQNIIPRASLAYENLTLYALECTMNILRLQREGSVVHLQRLIFVLGEYIVRNPFYTTFPKLISKTLSVVSTLIKVCPNERIVAHRFDILSLILVTYDKCSQEDALNESILQQCKETISWLLNCDCAMGEQLSTLSKQPRFQLLFEFS

>YNL210W|MER1

MSNQHSPQPFCLDTKLVKLLEELQEGKQFNNKNIFPEKALYLKLALDYSFFRKNLLEFCVHLDKIKGVIRPNYDTIYILCLLEVDLLNLVFTDNILEICLPRFVSREDLRVFNNTFYTYHDNRLRILQEDFSQLFKKIKTKASVLCFTVEEIFLTNQEILPQNSTVAELQKSTNKVQTNGPQRHDFIVTLEIKLNKTQITFLIGAKGTRIESLREKSGASIKIIPISDKMTAHERNHPESVQQTILISGDLYSIALAVTSIESALITLDL

>YGR029W|ERV1 MKAIDKMTDNPPQEGLSGRKIIYDEDGKPCRSCNTLLDFQYVTGKISNGLKNLSSNGKLAGTGALTGEASELMPGSRTYRKVDPPDVEQLGRSSWTLLHSVAASYPAQPTDQQKGEMKQFLNIFSHIYPCNWCAKDFEKYIRENAPQVESREELGRWMCEAHNKVNKKLRKPKFDCNFWEKRWKDGWDE

>YKR085C|MRPL20

MIGRGVCCRSFHTAGSAWKQFGFPKTQVTTIYNKTKSASNYKGYLKHRDAPGMYYQPSESIATGSVNSETIPRSFMAASDPRRGLDMPVQSTKAKQCPNVLVGKSTVNGKTYHLGPQEIDEIRKLRLDNPQKYTRKFLAAKYGISPLFVSMVSKPSEQHVQIMESRLQEIQSRWKEKRRIAREDRKRRKLLWYQA

>YPL227C|ALG5 MRALRFLIENRNTVFFTLLVALVLSLYLLVYLFSHTPRPPYPEELKYIAIDEKGHEVSRALPNLNEHQDDEEIFLSVVIPSYNETGRILLMLTDAISFLKEKYGSRWEIVIVDDGSTDNTTQYCLKICKEQFKLNYEQFRIIKFSQNRGKGGAVRQGFLHIRGKYGLFADADGASKFSDVEKLIDAISKIETSSTDLKTTKPAVAIGSRAHMVNTEAVIKRSMIRNCLMYGFHTLVFIFGIRSIKDTQCGFKLFNRAAILKIFPYLHTEGWIFDVEILILAIRKRIQIEEIPISWHEVDGSKMALAIDSIKMAKDLVIIRMAYLLGIYRDNKKC

>YMR197C|VTI1 MSSLLISYESDFKTTLEQAKASLAEAPSQPLSQRNTTLKHVEQQQDELFDLLDQMDVEVNNSIGDASERATYKAKLREWKKTIQSDIKRPLQSLVDSGDRDRLFGDLNASNIDDDQRQQLLSNHAILQKSGDRLKDASRIANETEGIGSQIMMDLRSQRETLENARQTLFQADSYVDKSIKTLKTMTRRLVANKFISYAIIAVLILLILLVLFSKFK

>YER146W|LSM5 MSLPEILPLEVIDKTINQKVLIVLQSNREFEGTLVGFDDFVNVILEDAVEWLIDPEDESRNEKVMQHHGRMLLSGNNIAILVPGGKKTPTEAL

>YBR247C|ENP1 MARASSTKARKQRHDPLLKDLDAAQGTLKKINKKKLAQNDAANHDAANEEDGYIDSKASRKILQLAKEQQDEIEGEELAESERNKQFEARFTTMSYDDEDEDEDEDEEAFGEDISDFEPEGDYKEEEEIVEIDEEDAAMFEQYFKKSDDFNSLSGSYNLADKIMASIREKESQVEDMQDDEPLANEQNTSRGNISSGLKSGEGVALPEKVIKAYTTVGSILKTWTHGKLPKLFKVIPSLRNWQDVIYVTNPEEWSPHVVYEATKLFVSNLTAKESQKFINLILLERFRDNIETSEDHSLNYHIYRAVKKSLYKPSAFFKGFLFPLVETGCNVREATIAGSVLAKVSVPALHSSAALSYLLRLPFSPPTTVFIKILLDKKYALPYQTVDDCVYYFMRFRILDDGSNGEDATRVLPVIWHKAFLTFAQRYKNDITQDQRDFLLETVRQRGHKDIGPEIRRELLAGASREFVDPQEANDDLMIDVN
